# Unsupervised Learning of Persistent and Sequential Activity

**DOI:** 10.3389/fncom.2019.00097

**Published:** 2020-01-17

**Authors:** Ulises Pereira, Nicolas Brunel

**Affiliations:** ^1^Department of Statistics, The University of Chicago, Chicago, IL, United States; ^2^Department of Neurobiology, The University of Chicago, Chicago, IL, United States; ^3^Department of Neurobiology, Duke University, Durham, NC, United States; ^4^Department of Physics, Duke University, Durham, NC, United States

**Keywords:** unsupervised learning, persistent activity, sequential activity, synaptic plasticity, Hebbian plasticity, homeostatic plasticity

## Abstract

Two strikingly distinct types of activity have been observed in various brain structures during delay periods of delayed response tasks: Persistent activity (PA), in which a sub-population of neurons maintains an elevated firing rate throughout an entire delay period; and Sequential activity (SA), in which sub-populations of neurons are activated sequentially in time. It has been hypothesized that both types of dynamics can be “learned” by the relevant networks from the statistics of their inputs, thanks to mechanisms of synaptic plasticity. However, the necessary conditions for a synaptic plasticity rule and input statistics to learn these two types of dynamics in a stable fashion are still unclear. In particular, it is unclear whether a single learning rule is able to learn both types of activity patterns, depending on the statistics of the inputs driving the network. Here, we first characterize the complete bifurcation diagram of a firing rate model of multiple excitatory populations with an inhibitory mechanism, as a function of the parameters characterizing its connectivity. We then investigate how an unsupervised temporally asymmetric Hebbian plasticity rule shapes the dynamics of the network. Consistent with previous studies, we find that for stable learning of PA and SA, an additional stabilization mechanism is necessary. We show that a generalized version of the standard multiplicative homeostatic plasticity (Renart et al., 2003; Toyoizumi et al., 2014) stabilizes learning by effectively *masking* excitatory connections during stimulation and *unmasking* those connections during retrieval. Using the bifurcation diagram derived for fixed connectivity, we study analytically the temporal evolution and the steady state of the learned recurrent architecture as a function of parameters characterizing the external inputs. Slow changing stimuli lead to PA, while fast changing stimuli lead to SA. Our network model shows how a network with plastic synapses can stably and flexibly learn PA and SA in an unsupervised manner.

## Introduction

Selective persistent activity (PA) has been observed in many neurophysiological experiments in primates performing delayed response tasks, in which the identity or spatial location of a stimulus must be maintained in working memory, in multiple cortical areas, including areas in the temporal lobe (Fuster et al., 1982; Miyashita, 1988; Miyashita and Chang, 1988; Sakai and Miyashita, 1991; Nakamura and Kubota, 1995; Miller et al., 1996; Naya et al., 1996; Erickson and Desimone, 1999), parietal cortex (Koch and Fuster, 1989; Chafee and Goldman-Rakic, 1998), and prefrontal cortex (Fuster and Alexander, 1971; Funahashi et al., 1989, 1990, 1991; Miller et al., 1996). More recently, selective persistent activity has also been observed in mice (Guo et al., 2014; Liu et al., 2014; Inagaki et al., 2019) as well as flies (Kim et al., 2017). It has been hypothesized that PA represents the mechanism at a network level of the ability to hold an item in working (*active*) memory for several seconds for behavioral demands. Theoretical studies support the hypothesis that persistent activity is caused by recurrent excitatory connections in networks of heavily interconnected populations of neurons (Amit et al., 1994; Durstewitz et al., 2000; Wang, 2001; Brunel, 2005). In these models, PA is represented as a fixed point attractor of the dynamics of a network that has multiple stable fixed points. The connectivity matrix in such models has a strong degree of symmetry, with strong recurrent connections between sub-groups of neurons which are activated by the same stimulus. This connectivity matrix can be learned by modifying recurrent connections in a network according to an unsupervised Hebbian learning rule (Mongillo et al., 2005; Litwin-Kumar and Doiron, 2014; Zenke et al., 2015).

Sequential activity (SA) has been also observed across multiples species in a number of behaviors such as spatial navigation (Foster and Wilson, 2006; Harvey et al., 2012; Grosmark and Buzsáki, 2016) and bird song generation (Hahnloser et al., 2002; Amador et al., 2013; Okubo et al., 2015). Furthermore, a large body of experimental evidence shows that SA can be learned throughout experience (Okubo et al., 2015; Grosmark and Buzsáki, 2016). Several theoretical network models have been able to produce SA (Amari, 1972; Kleinfeld and Sompolinsky, 1988; Abeles, 1991; Diesmann et al., 1999; Izhikevich, 2006; Liu and Buonomano, 2009; Fiete et al., 2010; Waddington et al., 2012; Cannon et al., 2015). In these models, the connectivity contains a feedforward structure—neurons active at a given time in the sequence project in a feedforward manner to the group of neurons which are active next. From a theoretical stand point, the mechanism to generate SA is fundamentally different from the one that generates PA. While SA usually corresponds to a path in the state space of the network, PA is identified as a fixed point attractor. Thus, SA has an inherent transient nature while PA is at least linearly stable in a dynamical system sense.

The question of how sequential activity can be learned in networks with plastic synapses has received increased interest in recent years. The models investigated can be roughly divided in two categories: models with supervised and unsupervised plasticity rules. In models with supervised plasticity rules, the synapses are updated according the activity of the network and an *error signal* that carries information about the difference between the current network dynamics and the one that it is expected to learn by the network (Sussillo and Abbott, 2009; Laje and Buonomano, 2013; Memmesheimer et al., 2014; Rajan et al., 2016). In models with unsupervised plasticity rules, sequential dynamics is shaped by external stimulation without an error signal (Jun and Jin, 2007; Liu and Buonomano, 2009; Fiete et al., 2010; Waddington et al., 2012; Okubo et al., 2015; Veliz-Cuba et al., 2015). In those models SA is generated spontaneously, and the temporal statistics of the stimulation shapes the specific timing of the sequences.

Both experimental and theoretical work therefore suggest that neural networks in the brain are capable to learn PA and SA. One unresolved issue is whether the learning rules used by brain networks to learn PA are fundamentally different than the ones used to learn SA, or whether the same learning rule can produce both, depending on the statistics of the inputs to the network. Learning rules employed in theoretical studies to learn PA typically do not contain any temporal asymmetry, while rules used to learn SA need to contain such a temporal asymmetry.

Here, we hypothesize that a single learning rule is able to learn both, depending on the statistics of the inputs. We investigate what are the conditions for the plasticity mechanisms and external stimulation to learn PA or SA using unsupervised plasticity rules. We consider a model composed of multiple populations of excitatory neurons, each activated by a distinct stimulus. We consider a sequential stimulation protocol in which each population of neurons is stimulated one at a time, one after the other. This protocol is characterized by two parameters, the duration of stimulus presentations and the time interval between stimulations. This simple setting allows us to explore between the extremes of isolated stimulations with short or large duration and sequential stimulations close or far apart temporally. We use a rate model to describe the activity of populations of neurons (Wilson and Cowan, 1972). The connectivity in this model represents the average of the synaptic connections between populations of neurons, allowing to investigate at a mesoscopic level the learning mechanisms of PA and SA. This model has the advantage of analytical tractability.

This paper is organized as follows: We first characterize the types of possible dynamics observed in network with both feedforward and recurrent connections, in the space of possible (fixed) connectivities. We then show that a network with plastic connections described by a unsupervised temporally asymmetric Hebbian plasticity rule stimulated sequentially does not stably learn PA and SA. We then explore two types of stabilization mechanisms: (1) synaptic normalization; (2) a generalized version of the standard multiplicative homeostatic plasticity (Renart et al., 2003; Toyoizumi et al., 2014). We show that when a synaptic normalization mechanism is included, PA and SA cannot be learned stably during sequential stimulation. However, the addition of a generalized homeostatic learning rule leads to successful learning of PA or SA by weakening the excitatory synaptic weights during stimulation, effectively *masking* the connectivity shaped by Hebbian learning. After stimulation, the learned connectivity structure is *unmasked* by the strengthening of the synaptic connections in a multiplicative fashion. PA or SA is learned depending on the temporal parameters of external inputs, and the learning can be characterized analytically as a dynamical system in the space of fixed connectivities parametrized by the stimulus parameters.

## 1. Methods

### 1.1. Networks With Fixed Connectivity

We first consider two different *n* population rate models that share in common two connectivity motifs that have been classically considered a distinctive feature of PA and SA respectively: recurrent and feedforward connections. The two network models considered are: (1) *n* excitatory populations; (2) *n* excitatory populations with shared inhibition. The strength of the recurrent and feedforward connections are *w* and *s*, respectively. We used the current based version of the widely used firing rate model, which is equivalent to its rate based version (Miller and Fumarola, 2012) with three different non-linear transfer functions.

#### 1.1.1. Network of Excitatory Neurons

**Figure 1:**
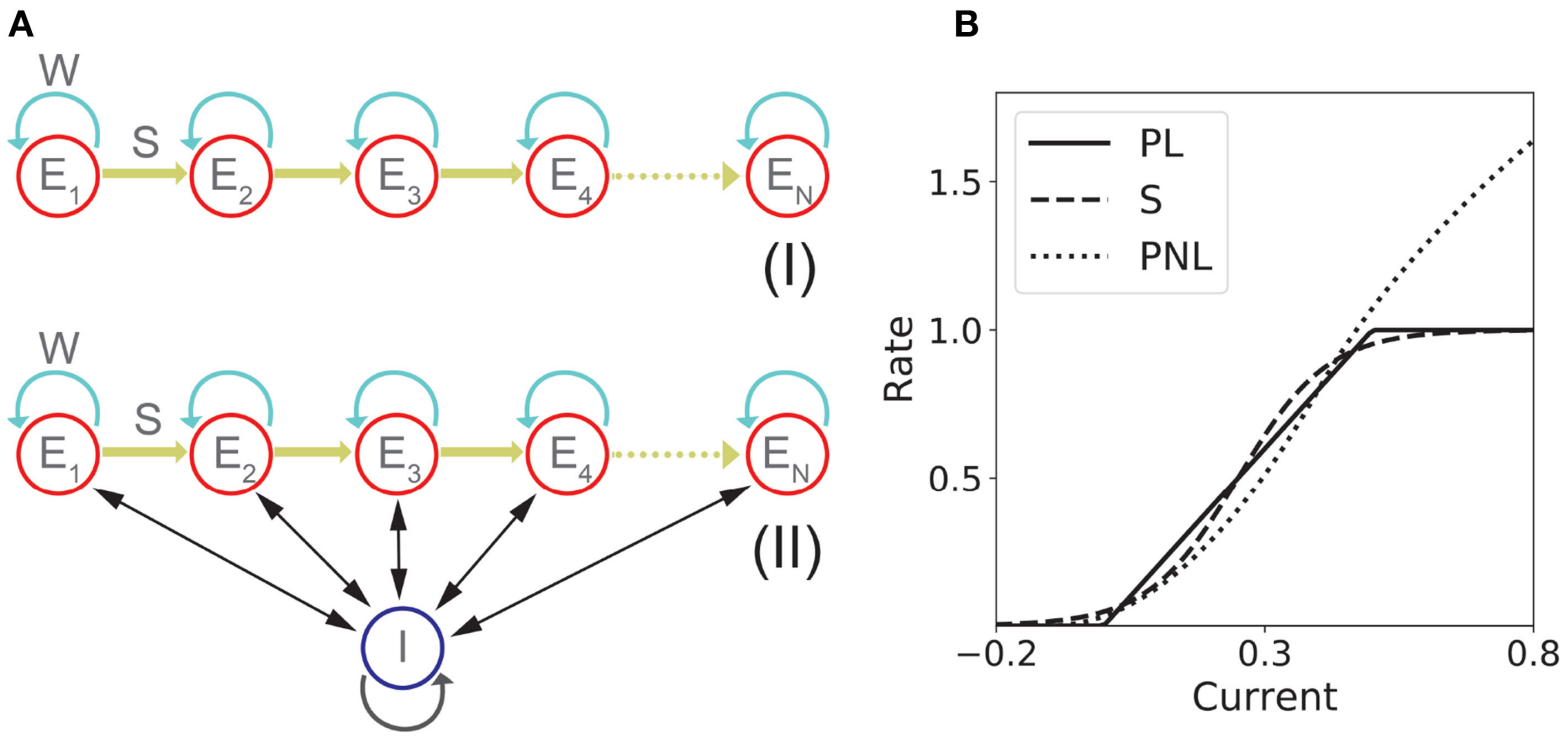
Network models with fixed connectivity. **(A)** Two models of recurrent and feedforward connected populations: (I) pure excitatory; (II) excitatory with shared inhibition. **(B)** Transfer Functions: piecewise linear (PL), sigmoidal (S), and piecewise non-linear (PNL).

The network consists in *n* excitatory populations connected by recurrent and feedforward connections with strength *w* and *s*, respectively as it is shown in Figure 1AI. The dynamics is given by:

(1) $$\begin{array}{l} \tau \frac{du_{1}}{dt}=I_{1}-u_{1}+w\phi (u_{1})\\ \tau \frac{du_{i}}{dt}=I_{i}-u_{i}+w\phi (u_{i})+s\phi (u_{i-1})\;i=2,\ldots ,n \end{array}$$

where *u*_*i*_ and *I*_*i*_ correspond to the synaptic and the external input to population *i* respectively, τ is the characteristic time scale for excitatory populations, and ϕ(*u*) is the current to average firing rate transfer function (or f-I curve). The resulting average firing rates are denoted by *r*_*i*_ ≡ ϕ(*u*_*i*_).

#### 1.1.2. Network of Excitatory Neurons With Shared Inhibition

The network consists in *n* excitatory populations connected as in section 1.1.1, and a single inhibitory population fully connected with the excitatory populations. A schematic of the network architecture is shown in Figure 1AII. Assuming a linear inhibitory transfer function, the dynamics of the network is given by:

(2) $$\begin{array}{l} \;\tau \frac{du_{1}}{dt}=I_{1}-u_{1}+w\phi (u_{1})-w_{EI}u_{I}\\ \;\tau \frac{du_{i}}{dt}=I_{i}-u_{i}+w\phi (u_{i})+s\phi (u_{i-1})-w_{EI}u_{I}\;i=2,\ldots ,n\\ \tau_{I}\frac{du_{I}}{dt}=-u_{I}+w_{IE}\sum_{j=1}^{n}\phi (u_{j}), \end{array}$$

where *w*_*EI*_ is the average inhibitory synaptic strength from inhibitory to excitatory populations, *w*_*IE*_ the average inhibitory synaptic strength from excitatory to inhibitory populations and τ_*I*_ the characteristic time scale of the inhibitory population. When τ_*I*_ ≪ τ, then $u_{I}\approx w_{IE}\overset{N}{\underset{i=1}{\sum }}\phi \left(u_{i}\right)$ and Equation (2) becomes

(3) $$\begin{array}{l} \tau \frac{du_{1}}{dt}=I_{1}-u_{1}+w\phi (u_{1})-\frac{w_{I}}{n}\sum_{j=1}^{n}\phi (u_{j})\\ \tau \frac{du_{i}}{dt}=I_{i}-u_{i}+w\phi (u_{i})+s\phi (u_{i-1})\\ \;-\frac{w_{I}}{n}\sum_{j=1}^{n}\phi (u_{j})\;i=2,\ldots ,n, \end{array}$$

where *w*_*I*_ ≡ *nW*_*EI*_*W*_*IE*_. See Figure S1 for the agreement between the full model described in Equation (2) and its approximation in Equation (3).

### 1.2. Transfer Functions

For the fixed connectivity part of this study we used three different families of transfer functions. The sigmoidal transfer function is described by

(4) $$\phi (u)=\frac{1}{2}\left(1+\tanh[a(u+b)]\right).$$

This is a saturating monotonic function of the total input, and represents a normalized firing rate. This transfer function has been widely used in many theoretical studies in neuroscience (Ermentrout and Terman, 2010; Gerstner et al., 2014), and has the advantage to be smooth. Furthermore, we have recently shown that such transfer functions provide good fits to *in vivo* data (Pereira and Brunel, 2018).

The second transfer function considered is piecewise linear:

(5) $$\phi (u)=\{\begin{array}{ll} 0 & \text{if }\theta >u\\ \nu (u-\theta ) & \text{if }\theta \leq u\leq u_{c}\\ \nu (u_{c}-\theta ) & u_{c}<u. \end{array}$$

This is a piecewise linear approximation of the sigmoidal transfer function. The main advantage of this transfer function is its analytical tractability—the non-linear dynamics of a network with such a transfer function can be analyzed as a piecewise linear dynamical system.

The third transfer function used in this work is piecewise non-linear (Brunel, 2003)

(6) $$\phi (u)=\{\begin{array}{ll} 0 & \text{if }\tilde{\theta }>u\\ \tilde{\nu }{\left(\frac{u-\tilde{\theta }}{{\tilde{u}}_{c}-\tilde{\theta }}\right)}^{2} & \text{if }\tilde{\theta }\leq u\leq {\tilde{u}}_{c}\\ 2\tilde{\nu }\sqrt{\frac{u-\tilde{\theta }}{{\tilde{u}}_{c}-\tilde{\theta }}-\frac{3}{4}} & {\tilde{u}}_{c}<u. \end{array}$$

This transfer function combines several features that are present in more realistic spiking neuron models and/or real neurons: a supralinear region at low rates, described by a power law (Roxin et al., 2011), and a square root behavior at higher rates, as expected in neurons that exhibit a saddle-node bifurcation to periodic firing (Ermentrout and Terman, 2010). Examples of these three transfer functions are shown in Figure 1B.

### 1.3. Sequential Stimulation

During the learning protocol excitatory populations are stimulated sequentially once at a time for a period *T* and a time delay Δ. The stimulation can be implemented as a sequence of vectors presented to the entire the network (i.e., $I{\vec{e}}_{1},I{\vec{e}}_{2},\ldots ,I{\vec{e}}_{n}$), each vector corresponds to the canonical base in ℝ^*n*^ scaled by a stimulation amplitude *I*. This sequence of stimulation is repeated *k* times. To prevent a concatenation between the first and the last population stimulated, the period between each repetition *k* is much longer than *T* and Δ and any time constant of the network. Each stimulus in the sequence has the same magnitude, that is larger than the learning threshold (i.e., *r*_*w*_ < *I*). A schematic diagram of the stimulation protocol is described in section 2.2.

### 1.4. Temporally Asymmetric Hebbian Plasticity Rule

When a temporally asymmetric Hebbian plasticity rule is included (see sections 2.2–2.5 in Results), the dynamics of excitatory-to-excitatory connectivity obeys

(7) $$\frac{dW_{i,j}}{dt}=\frac{w_{max}f[r_{i}(t)]g[r_{j}(t-D)]-W_{i,j}}{\tau_{w}[r_{i}(t),r_{j}(t-D)]},$$

where *f*(*r*) and *g*(*r*) are sigmoidal functions given by

(8) $$g(r)=\frac{1}{2}\left(1+\tanh\left[a_{\text{pre}}(r-b_{\text{pre}})\right]\right)$$

(9) $$f(r)=\frac{1}{2}\left(1+\tanh\left[a_{\text{post}}(r-b_{\text{post}})\right]\right).$$

They describe the dependence of the learning rule on post- and pre-synaptic firing rates, respectively [i.e., their dependence on ϕ(*u*_*i*_) and ϕ(*u*_*j*_)], and are bounded by zero for small or negative values of the population synaptic current, and by one for large values (see Figures 4A,B). Here *w*_*max*_ is the maximal synaptic efficacy; *D* is a temporal delay; and τ_*w*_ is an activity-dependent time constant of the plasticity rule. The learning time scale is given by

(10) $$\tau_{w}[r_{i}(t),r_{j}(t-D)]=\{\begin{array}{ll} \infty & \text{if }r_{i}<r_{w}\text{ and }r_{j}<r_{w}\\ T_{w} & \text{if }r_{w}\leq r_{i}\text{ or }r_{w}\leq r_{j}. \end{array}$$

(see dashed line in Figures 4A–C) and time scale respectively. The time scale *T*_*w*_ is chosen to be several order of magnitude slower than the population dynamics (see Table S3). When pre- and post-synaptic firing rates are below a plasticity threshold *r*_*w*_, the activity-dependent time constant τ_*w*_ becomes infinite, and therefore no plasticity occurs. When pre and/or post-synaptic firing rates are above *r*_*w*_, then the activity-dependent time constant τ_*w*_ is equal to *T*_*w*_, and plasticity is ongoing. Thus, with this rule strong, long (i.e., large *T*) and/or frequent enough (i.e., short Δ) stimuli produce lasting modifications in the synaptic weights. When *w*_*max*_*f*[*r*_*i*_(*t*)]*g*[*r*_*j*_(*t*−*D*)] < 𝕎_*i, j*_, the corresponding synaptic weight tend to decrease, and therefore LTD takes place. For a fixed high pre-synaptic firing rate, LTD occurs when post-synaptic firing rates are low, similar to learning rules such as the BCM rule (Bienenstock et al., 1982) and Hebbian rules used in recurrent networks (Mongillo et al., 2003). Likewise, for a fixed high post-synaptic firing rate, LTD also occurs in an intermediate and low region of pre-synaptic firing rates. Conversely, when 𝕎_*i, j*_ < *w*_*max*_*f*[*r*_*i*_(*t*)]*g*[*r*_*j*_(*t* − *D*)], the corresponding synaptic weight tend to increase, and LTP takes place.

In our model, we assume the activity of each population represents the average activity over many neurons (of the order of tens of thousands). Likewise, synaptic couplings are assumed to represent averages over very large numbers of synapses connecting two populations (or one population to itself). Therefore, the learning parameters (e.g., *D* and *T*_*w*_) represent average quantities over a large number of synapses. We expect the variability of these parameters to disappear in the limit of large number synaptic couplings.

### 1.5. Synaptic Normalization

When a synaptic normalization mechanism is included (see section 2.3 in Results), in addition to the Hebbian plasticity rule described in section 1.4, in our network simulations, at each time step we subtracted the average synaptic change to each incoming synapse to a given population. This average is taken over all the incoming synapses to a particular neuron. This simulation scheme ensures that the sum of the incoming synaptic weights to each population remains constant, i.e.,

(11) $$\sum_{j=1}^{n}W_{i,j}=C\;i=1,2,\ldots ,n.$$

### 1.6. Generalized Homeostatic Plasticity Rule

We implement a modified version of the multiplicative homeostatic rule proposed in Renart et al. (2003) and Toyoizumi et al. (2014) (see sections 2.4 and 2.5 in Results). The rule is implemented in addition to the Hebbian plasticity rule described in the section 1.4. In this rule a homeostatic variable *H*_*i*_ slowly controls the firing rate of neuron *i* by scaling its synaptic weights multiplicatively. The synaptic weights will be given by

(12) $$W_{i,j}(t)=H_{i}(t)W_{i,j}(t).$$

The variable 𝕎_*i, j*_(*t*) is governed by the Hebbian plasticity rule described by Equations (7–10). The dynamics for *H*_*i*_ is given by

(13) $$\tau_{H}{\dot{H}}_{i}=\left(1-\frac{r_{i}(t)}{r_{0}}\right)H_{i}-H_{i}^{2},$$

where *r*_0_ = ϕ(*u*_0_) is a parameter that controls the *average* firing rate of population *i* and τ_*H*_ is the characteristic time scale of the learning rule. Note that because of the quadratic term in the r.h.s. of Equation (13), this rule does not in general keep the firing rates at a fixed value, and therefore this rule is not strictly speaking homeostatic. Therefore, we term this rule generalized homeostatic plasticity rule, since this rule is a generalization of the standard multiplicative homeostatic plasticity rule in Renart et al. (2003) and Toyoizumi et al. (2014). As in the standard rule, for strong inputs, the homeostatic variables of this rule decrease to values that are smaller than 1, scaling down the excitatory connections and *masking* synaptic changes learned via Hebbian plasticity. However, unlike the standard rule, after stimulation, the homeostatic variables return to values $H_{i}\sim O\left(1\right)$, and the synaptic changes learned via Hebbian plasticity are *unmasked*. See section 2.4 and section 5 in the Supplementary Material for a detailed analysis of this rule.

### 1.7. Learning Dynamics Under Noisy Stimulation

In the last section of the Results, we include noise in the population dynamics in order to asses the robustness of the learning process (see section 2.5 in Results). The equations used to describe the dynamics of the network with Hebbian and generalized homeostatic plasticity are given by

(14) $$\begin{array}{l} \;\tau {\dot{u}}_{i}=-u_{i}+\sigma \eta_{i}+I_{i}+\sum_{j=1}^{n}H_{i}W_{i,j}r_{j}-\frac{W_{I}}{n}\sum_{i=1}^{n}\phi (u_{i})\\ {\dot{\;W}}_{i,j}=\frac{w_{max}f[r_{i}(t))]g[r_{j}(t-D)]-W_{i,j}}{\tau_{w}(r_{i}(t),r_{j}(t-D))}\\ \tau_{H}{\dot{H}}_{i}=\left(1-\frac{r_{i}(t)}{r_{0}}\right)H_{i}-H_{i}^{2}, \end{array}$$

where *r*_*i*_(*t*) = ϕ(*u*_*i*_(*t*)) for *i* = 1, 2, …, *n* and η_*i*_ is a Gaussian white noise.

### 1.8. Code

Simulations were performed using code written in Python. A self-contained version of the code that reproduces all the figures in this paper is available in the GitHub repository: https://github.com/ulisespereira/Unsupervised.

## 2. Results

### 2.1. Persistent and Sequential Activity in Networks With Fixed Connectivity

To better understand the dependence of PA and SA generation on network connectivity, we consider first a simple *n* population rate model with fixed feedforward and recurrent connectivity (see Figure 1A). This architecture possesses the two connectivity motifs that have been classically considered the hallmarks of PA and SA—recurrent and feedforward connections—in a space of parameters that is low dimensional enough to be suitable for full analytical treatment. In this model, the dynamics of the network is characterized by the synaptic inputs *u*_*i*_ to each population of the network (*i* = 1, …, *n*) whose dynamics obey the system of ordinary differential equations in Equation (1). Note that we use here the *current based* formulation of the firing rate equations, that has been shown to be equivalent to the *rate based* formulation (Miller and Fumarola, 2012).

**Figure 2:**
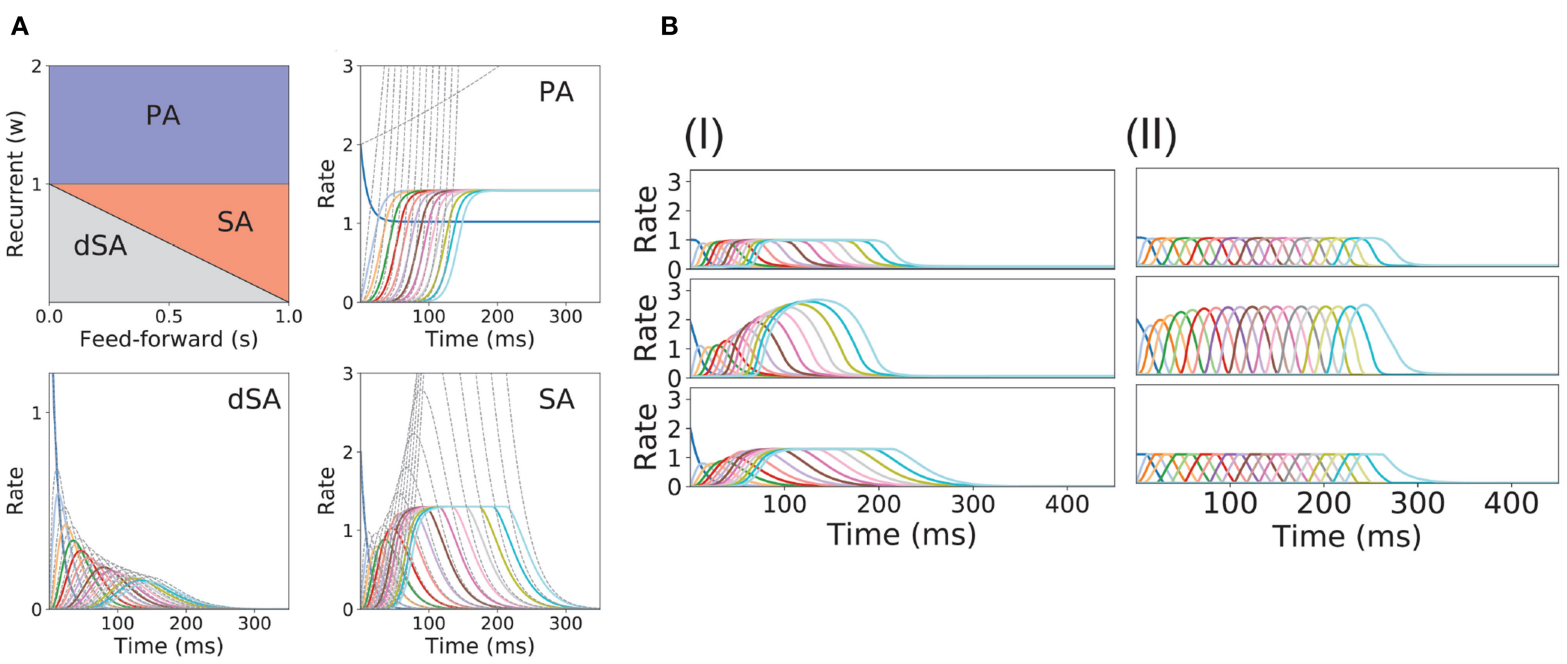
PA and SA generation in a network with fixed connectivity. **(A)** Phase diagram for model (I) using a piecewise linear transfer function (top-left plot) and examples of the dynamics corresponding to the three phases. Dashed lines correspond to the dynamics for the same network but using a linear transfer function. **(B)** SA generation for models (I) and (II) using sigmoidal (first row), piecewise non-linear (second row), and piecewise linear (third row) transfer functions. The parameters for the transfer functions are the same as the ones used in Figure 1B. Firing rates of excitatory populations are colored according to their position in the feedforward-recurrent connected excitatory network. Parameters used in **(A,B)** can be found in Table S1.

In this model, we identify the regions in the connectivity parameter space where SA, PA, or decaying sequences of activity (dSA) are generated. We start with a piecewise linear transfer function with slope ν, and compute the bifurcation diagram that gives the boundaries for qualitatively different dynamics in the parameter space (see Figure 2A and section 2 in the Supplementary Material for mathematical details). We find that robust SA can be generated provided recurrent connections are smaller than the inverse of the slope ν, and the feedforward connections are strong enough, *w* < 1/ν < *w*+*s*. For large values of *w* (*w* > 1/ν), the dynamics converge to a fixed point where 0 ≤ *p* ≤ *n* populations are in a high rate state, where *p* depends on the initial conditions. When both recurrent and feedforward connections are weak enough (i.e., *w*+*s* < 1/ν) the activity decays to zero firing rate fixed point, after a transient in which different populations are transiently activated—a pattern which we term decaying sequence of activity or dSA.

This picture is qualitatively similar when other types of non-linear transfer functions are used (see Methods and Figure 1B for the transfer functions used in this paper). The saturation non-linearity of the transfer function is key to generate long lasting (non-attenuated) SA even when the number of populations is large. In a linear network, sequential activity would increase without bound for an increasing number of populations participating in the SA (see Figure 2A, dashed lines and section 2 in the Supplementary Material for mathematical details). During sequential activity, each population is active for a specific time interval. We used the analytical solution of the linearized system (see Equation S3) to show that the duration of this active interval scales as the squared root of the position of the population along the sequence. This implies that for long lasting SA the fraction of active populations will increase with time (see Figure 2A). This feature is not consistent with experimental evidence that shows that the width of the bursts of activity along the sequence is approximately constant in time (Hahnloser et al., 2002; Harvey et al., 2012). In the model, we can prevent this phenomenon by including negative feedback mechanisms to our network architecture as global inhibition (see Figure 1AII). We found that in both cases the network robustly generates PA and SA in which the fraction of active populations is approximately constant in time. These results were also qualitatively similar when different saturation non-linearities in the transfer function were considered (see Figure 2B).

**Figure 3:**
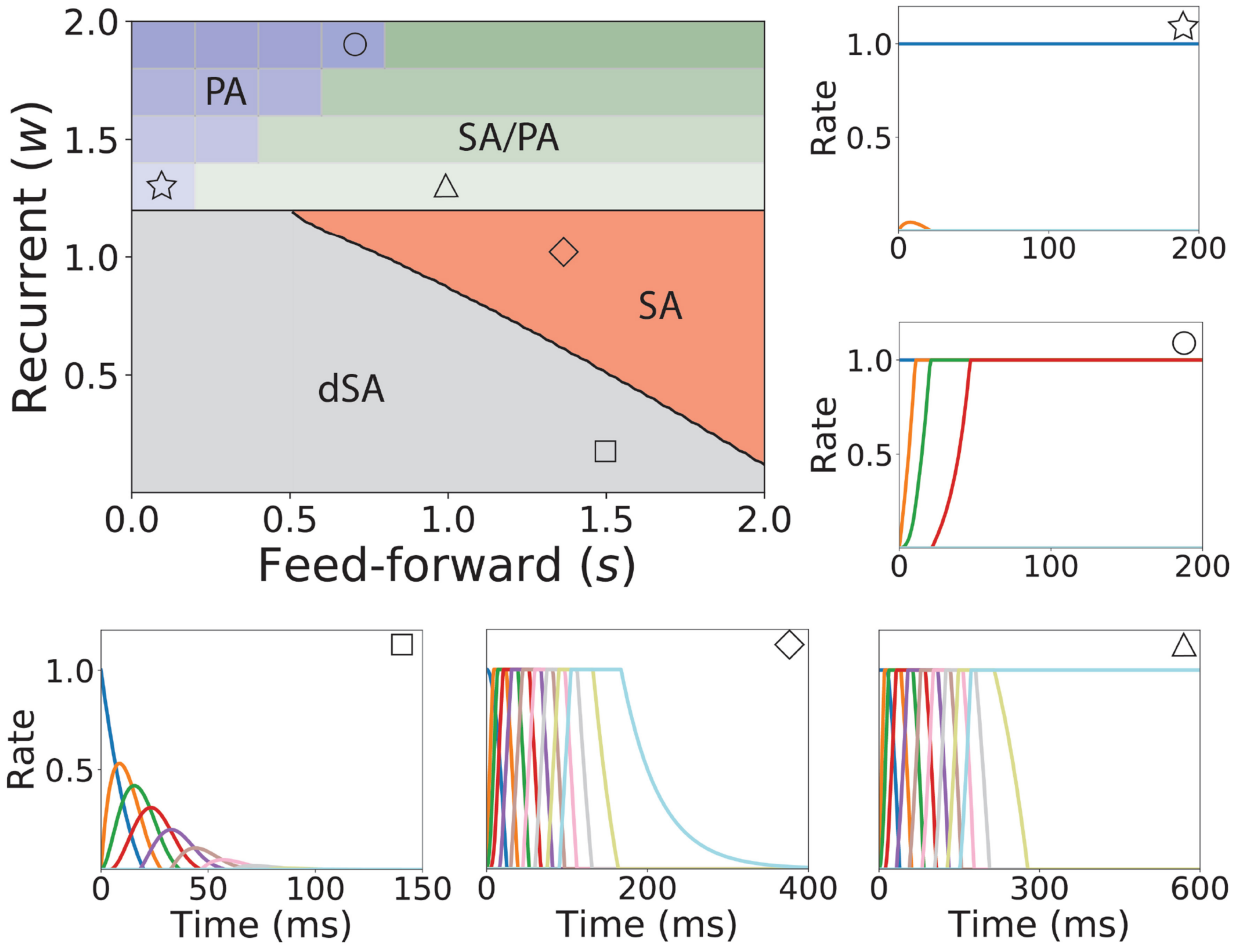
Bifurcation diagram for feedforward-recurrent connected network of excitatory populations with shared inhibition. Top left plot: Bifurcation diagram in the *s*-*w* plane, showing qualitatively different regions: dSA (gray), SA (red), SA/PA (green), and PA (blue). The PA region is divided in sub-regions which are distinguished by the maximum and minimum number of populations active during PA (see text). The SA/PA region is also subdivided into sub-regions characterized by a different number of the maximum number of populations active in PA at the end of the sequence. Regions are separated by black lines and sub-regions are separated by gray lines. Five plots encompassing the bifurcation diagram show examples of the dynamics observed in its four qualitatively different regions. Initial condition: first population active at the maximum rate, while the rest is silent. The location in the corresponding regions of the parameter space are indicated with the symbols on the top right of the surrounding plots. Parameters can be found in Table S2. Firing rates of excitatory populations are colored as in Figure 2.

We now turn our attention to the network of excitatory neurons with global inhibition (Figure 1AII), since inhibition is likely to be the dominant source of negative feedback in local cortical circuits. Inhibitory interneurons are typically faster than excitatory neurons (McCormick et al., 1985). For the sake of simplicity we set the inhibitory population dynamics as instantaneous compared with the excitatory timescale. Our numerical simulations confirm that this approximation preserves all the qualitative features of the dynamics with finite inhibitory time constants, up to values of τ_*I*_ = 0.5τ (see Figure S1). Using this approximation, the connectivity of the network is equivalent to a recurrent and feedforward architecture plus a uniform matrix whose elements are *w*_*I*_ ≡ *nw*_*EI*_*w*_*IE*_. We obtained the bifurcation diagram for such a network with a piecewise linear transfer function (see section 4 in the Supplementary Material). This new bifurcation diagram shows qualitative differences with the pure excitatory network bifurcation diagram (see Figure 3). First, a qualitatively different behavior arises, where SA ends in persistent activity (region SA/PA). Second, the PA region breaks down in *n*(*n* + 1)/2 square regions of size *w*_*I*_/*n* × *w*_*I*_/*n*. Each region is characterized by a minimum and maximum number of populations active during PA. The lower left corner of each squared region is $\left(i_{min}\left(\frac{w_{I}}{n}\right),1+i_{max}\left(\frac{w_{I}}{n}\right)\right)$ with *i*_*min*_, *i*_*max*_ = 1, 2, …, *n* (see Figure 3, different regions in graded blue), where *i*_*min*_ and *i*_*max*_ correspond to the minimum and maximum number of population active during PA within this squared region when just the first population is initialized in the active state (Figure 3 top and middle right plots). Therefore, the number of possible patterns of PA increases with the strength of the recurrent connections and decreases with strength of the feedforward connections. On the other hand, the SA/PA is divided in *n* qualitatively different rectangular regions of size $\left(\frac{w_{I}}{n}\right)\times \left[1-j_{SA/PA}\left(\frac{w_{I}}{n}\right)\right]$ with *j*_*SA*/*PA*_ = 1, 2, …, *n*, where *j*_*SA*/*PA*_ corresponds to the number of populations that ends in PA after SA elicited by stimulating the first population in the sequence (Figure 3 bottom right plot). Then for a given strength of the recurrent connectivity w^*^ above $1+\left(\frac{w_{I}}{n}\right)$, the critical feedforward strength *s*_*c*_ that separates the PA and SA/PA regions is

(15) $$s_{c}=\frac{w_{I}}{n}\left\lceil \frac{\left(w^{*}-1-\frac{w_{I}}{n}\right)n}{w_{I}}\right\rceil ,$$

where ⌈·⌉ is the ceiling function. Similarly, for a given strength of the feedforward connection s^*^ above $\frac{w_{I}}{n}$, the critical recurrent strength separating SA/PA and PA is

(16) $$w_{c}=\frac{w_{I}}{n}\left\lceil \frac{\left(s^{*}-\frac{w_{I}}{n}\right)n}{w_{I}}\right\rceil .$$

Lastly, we find that the SA region is shrunk compared with the pure excitatory network, and that the dSA region is wider.

### 2.2. Unsupervised Temporally Asymmetric Hebbian Plasticity Rule

**Figure 4:**
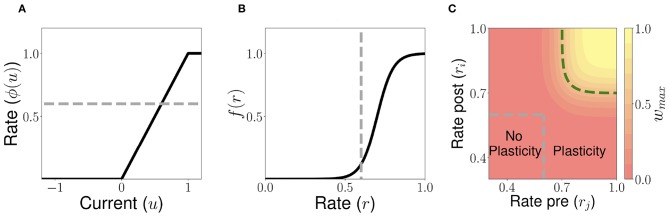
Unsupervised Hebbian learning rule. **(A)** Piecewise linear transfer function. The dashed gray horizontal line indicates the plasticity threshold *r*_*w*_. **(B)** Post-synaptic dependence on the rates of the stationary connectivity function, *f*(*r*). The vertical dashed gray line indicates the plasticity threshold. **(C)** Contour plot of the stationary connectivity function, 𝕎_*max*_*f*(*r*_*i*_)*g*(*r*_*j*_). The dashed gray box indicates the plasticity threshold. For *W*_*i,j*_ = 0.5*w*_*max*_ the red region below the dashed green line corresponds to LTD while the yellow region above it to LTP. Parameters can be found in Table S3.

Let us consider now a fully connected network of *n* excitatory populations with plastic synapses and global fixed inhibition. The plasticity rule for the excitatory-to-excitatory connectivity is described by Equation (7). Using this learning rule, with fixed pre- and post-activity, the connectivity tends asymptotically to a separable function of the pre- and post-synaptic activity. The functions *f*(*r*) and *g*(*r*) are bounded by zero for small or negative values of the population synaptic current, and by one for large values (see Figures 4A,B). This learning rule is a generalization of classic Hebbian rules like the covariance rule (Dayan and Abbott, 2001), with a non-linear dependence on both pre and post-synaptic firing rates.

The delay *D* in the learning rule leads to a temporal asymmetry (Blum and Abbott, 1996; Gerstner and Abbott, 1997; Veliz-Cuba et al., 2015). This delay describes the time it takes for calcium influx through NMDA receptors to reach its maximum (Sabatini et al., 2002; Graupner and Brunel, 2012). When this learning rule operates and the network is externally stimulated, the connectivity changes depending on the interaction of the input, the network dynamics and the learning rule. Due to the relaxational nature of Equation (7), for long times without external stimulation the connectivity matrix will converge to a stationary rank-1 matrix with entries of the form $f\left(r_{i}^{*}\right)g\left(r_{j}^{*}\right)$, where ${\vec{r}}^{*}=\phi \left({\vec{u}}^{*}\right)$ is the stationary firing rate vector, independent of all inputs presented in the past. Therefore, stimuli learned in the connectivity matrix will be erased by the background activity of the network for long times after stimulation. To prevent this inherent forgetting nature of the learning rule we introduce an activity-dependent plasticity time scale in Equation (10). Thus, when pre and/or post-synaptic currents are below a plasticity threshold *r*_*w*_, the time scale becomes infinite, and therefore no plasticity occurs. When both are above *r*_*w*_, then the time constant is given by *T*_*w*_ [see Equation (10) and Figure 4]. Lastly, the time scale *T*_*w*_ of these changes are chosen to be several order of magnitude slower than the population dynamics, consistent with the time it takes (~1 min or more) for plasticity to be induced in standard synaptic plasticity protocols (see e.g., Markram et al., 1997; Bi and Poo, 1998; Sjöström et al., 2001, but see Bittner et al., 2017). Importantly, the weight changes are proportional to the difference *w*_*max*_*f*[*r*_*i*_(*t*)]*g*[*r*_*j*_(*t*−*D*)]−𝕎_*i, j*_, which depends on both the pre and post-synaptic firing rates and the current value of the synaptic weight (see Equation 7). Typically, when both pre and post-synaptic firing rates are at intermediate values (see the red region in Figure 4C) the corresponding synapse undergoes LTD (weight strength tend to decrease), and when they are both high (see the yellow region in Figure 4C) it undergoes LTP (wight strength tend to increase). Therefore, the rule goes from no plasticity to LTD and then to LTP when both pre and post-synaptic firing rates increase (see diagonal direction in Figure 4C), which is reminiscent of results in pairing experiments (Ngezahayo et al., 2000).

**Figure 5:**
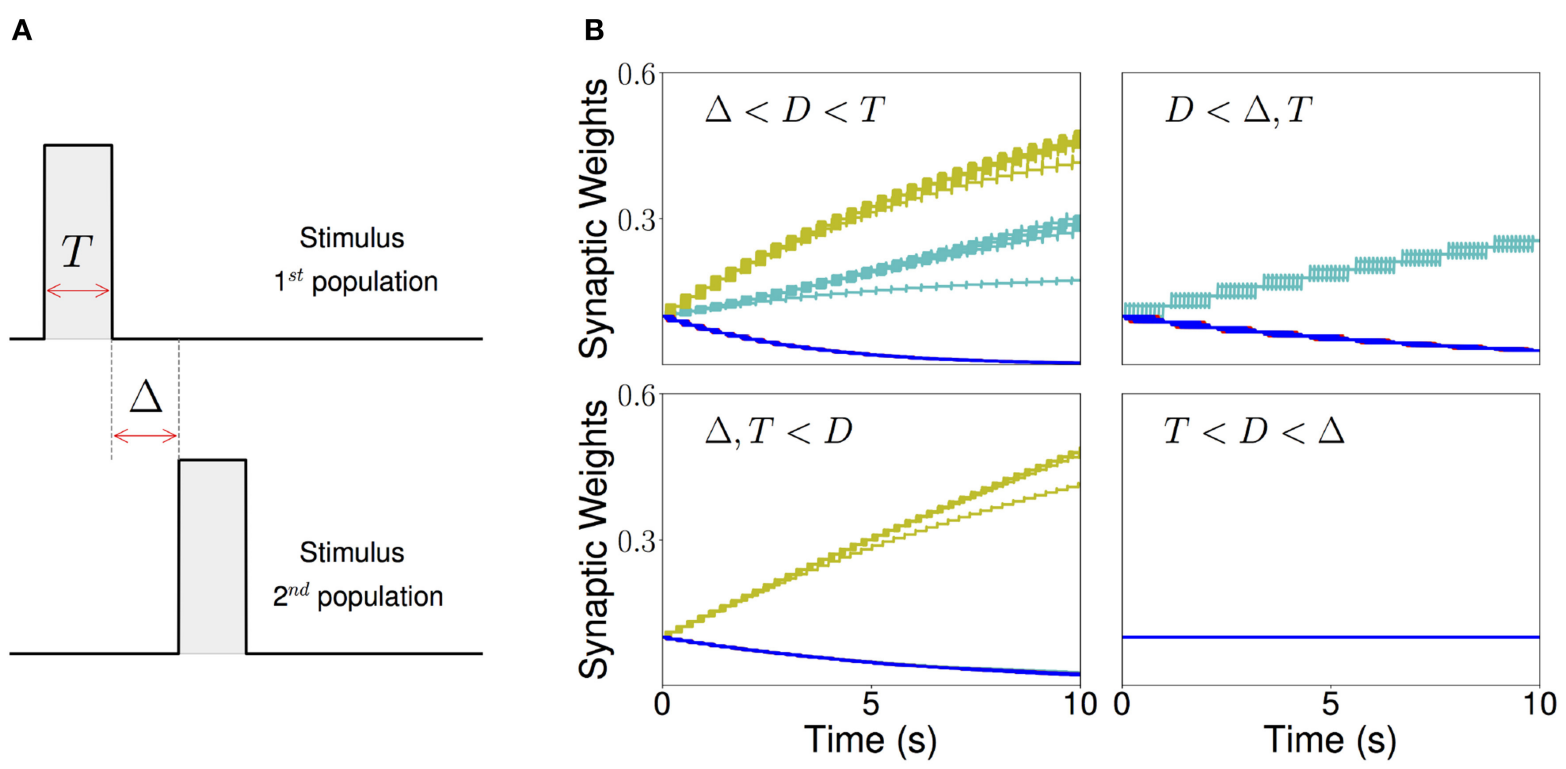
Sequential stimulation and initial synaptic weights dynamics. **(A)** Schematic diagram showing stimulation protocol for two populations. Population 1 is first stimulated for some time *T*. Then, after an inter-stimulation Δ time, population 2 is stimulated for the same duration *T*. **(B)** The weight dynamics for a network of excitatory populations with shared inhibition and a piecewise linear transfer function is shown for four different stimulation regimes. Top-left: Δ < *D* < *T*; top-right: *D* < Δ, *T*; bottom-left: *T*, Δ < *D*; bottom-right: *T* < *D* < Δ. Cyan: recurrent connections; Yellow/Green: feedforward; Blue: all other connections. Parameters can be found in Tables S3, S4.

Our goal is to understand the conditions for a sequential stimulation to lead the network dynamics to PA or SA, depending of the temporal characteristics of the stimulus, when this plasticity rule is introduced. Here we consider a simple stimulation protocol where each population in the network is stimulated sequentially one population at a time (see Figure 5A). In this protocol, population 1 is first stimulated for some time *T*. Then, after an inter-stimulation time Δ, population 2 is stimulated for the same duration *T*. The other populations are then stimulated one at a time (3, 4, …, *n*) using the same protocol. The amplitude of the stimulation is fixed such that the maximum of the current elicited in each population leads to a firing rate that is greater than the plasticity threshold of the learning rule. The time interval between each repetition of the sequence is much longer than *T* and Δ and any time constant of the network. When the duration of each stimulation is larger than the synaptic delay (i.e., *D* < *T*), recurrent connections increase, since the Hebbian term driving synaptic changes (*f*[*r*_*i*_(*t*)]*g*[*r*_*i*_(*t* − *D*)], where *i* is the stimulated population) becomes large after a time *D* after the onset of the presentation. When the inter-stimulation time is smaller that the synaptic delay (i.e., Δ < *D*), then the feedforward connections increase, since the Hebbian term driving synaptic changes (*f*[*r*_*i*+1_(*t*)]*g*[*r*_*i*_(*t* 𢄠 *D*)]) is large in some initial interval during presentation of stimulus *i*+1 (see also Herz et al., 1988).

As a result, there are four distinct regions of interest depending on the relative values of the Δ and *T* with respect to the synaptic delay *D*. When *T* is larger than the synaptic delay, and Δ is smaller than the synaptic delay, both recurrent and feedforward connections increase. When *T* is larger than the synaptic delay and Δ is much larger than *D*, only the recurrent connections increase. When Δ is smaller than the synaptic delay and *T* is much smaller, only the feedforward connections increase. Lastly, when Δ is larger and *T* is smaller than *D* no changes in the connectivity are observed. The initial temporal evolution of both recurrent and feedforward weights in representative examples of the four regions is presented in Figure 5B. We chose not to study the region corresponding to 2*T* + Δ < *D* here, which is a region where “feedforward” connections involving non-nearest neighbor populations can also increase during learning.

**Figure 6:**
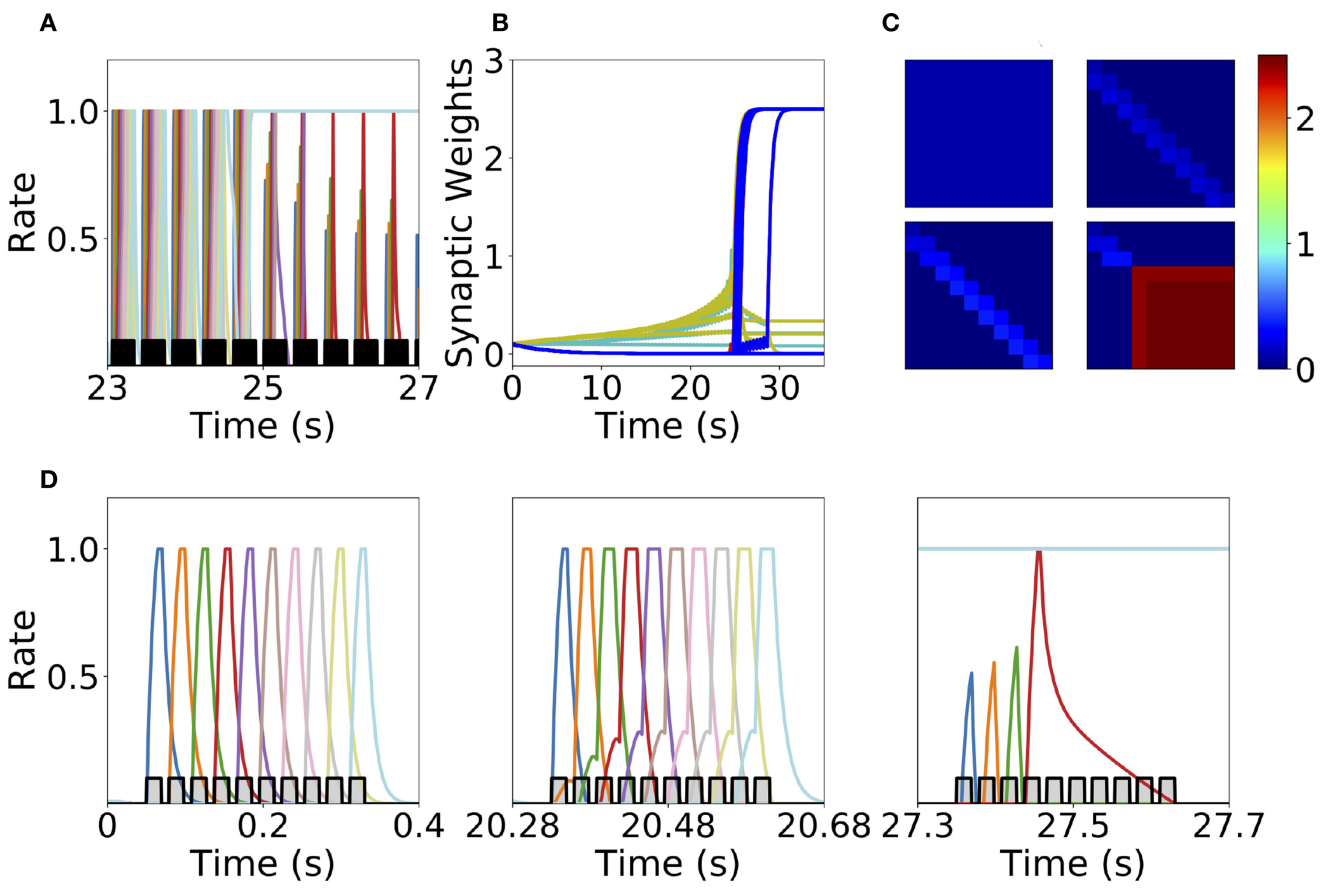
Runaway instability of the unsupervised Hebbian learning rule. **(A)** Population dynamics during 4 s of sequential stimulation with *T* = 19 ms and Δ = 10 ms. After about 25 s, the last six populations stimulated become active at maximal rates. **(B)** Synaptic weights dynamics during stimulation. Cyan: recurrent connections; Light Yellow/Green: feedforward; Red: feed-backward. **(C)** Connectivity matrix at different stimulation times. From left to right and from top to bottom: 0, 10, 20, and 30 s. **(D)** Three examples of population dynamics during a single sequential stimulation at 0, 20.28, and 27.3 s, respectively. Note the buildup of activity preceding each stimulus presentation because of the build-up in the feedforward connectivity at 20.28 s. In **(A,D)** the black and gray traces indicate a scaled version of the stimulus. Parameters can be found in Tables S3, S4. Firing rates of excitatory populations are colored according to their position in the sequential stimulation.

We found that this learning rule is in general unstable for long sequential stimulation when both feedforward and recurrent connections increase during the stimulation (i.e., Δ < *D* < *T*) to values large enough to produce persistent activity states. This is a consequence of the classic instability observed with Hebbian plasticity rules, where a positive feedback loop between the increase in synaptic connectivity and increase in firing rates leads to an explosive increase in both (Dayan and Abbott, 2001). Larger feedforward and recurrent connections lead to an increase in number of populations active at the same time during stimulation (see Figures 6A,D) which produce an increase of the overall connectivity by the synaptic plasticity rule (Figures 6B,C). This leads to an increase in the overall activity producing longer periods of PA during stimulation until a fixed point where many populations have high firing rates is reached, and the connectivity increases exponentially to its maximum value (see Figures 6B,C). By increasing the plasticity threshold, it is possible to increase the number of stimulations (and consequently the strength of the feedforward and recurrent connections) where the network's activity is stable. However, this does not solve the problem, since the instability on the weights eventually occurs but for a larger number of stimulations and stronger synaptic weights. In order to prevent this instability, we investigate in the next sections two different stabilization mechanisms: synaptic normalization and generalized homeostatic plasticity. Throughout this paper, for testing whether PA, SA, SA/PA, or dSA is learned, after sequential stimulation we stimulate the first population and then check whether the network recalls the corresponding type of activity (see Figure 3).

### 2.3. Synaptic Normalization

**Figure 7:**
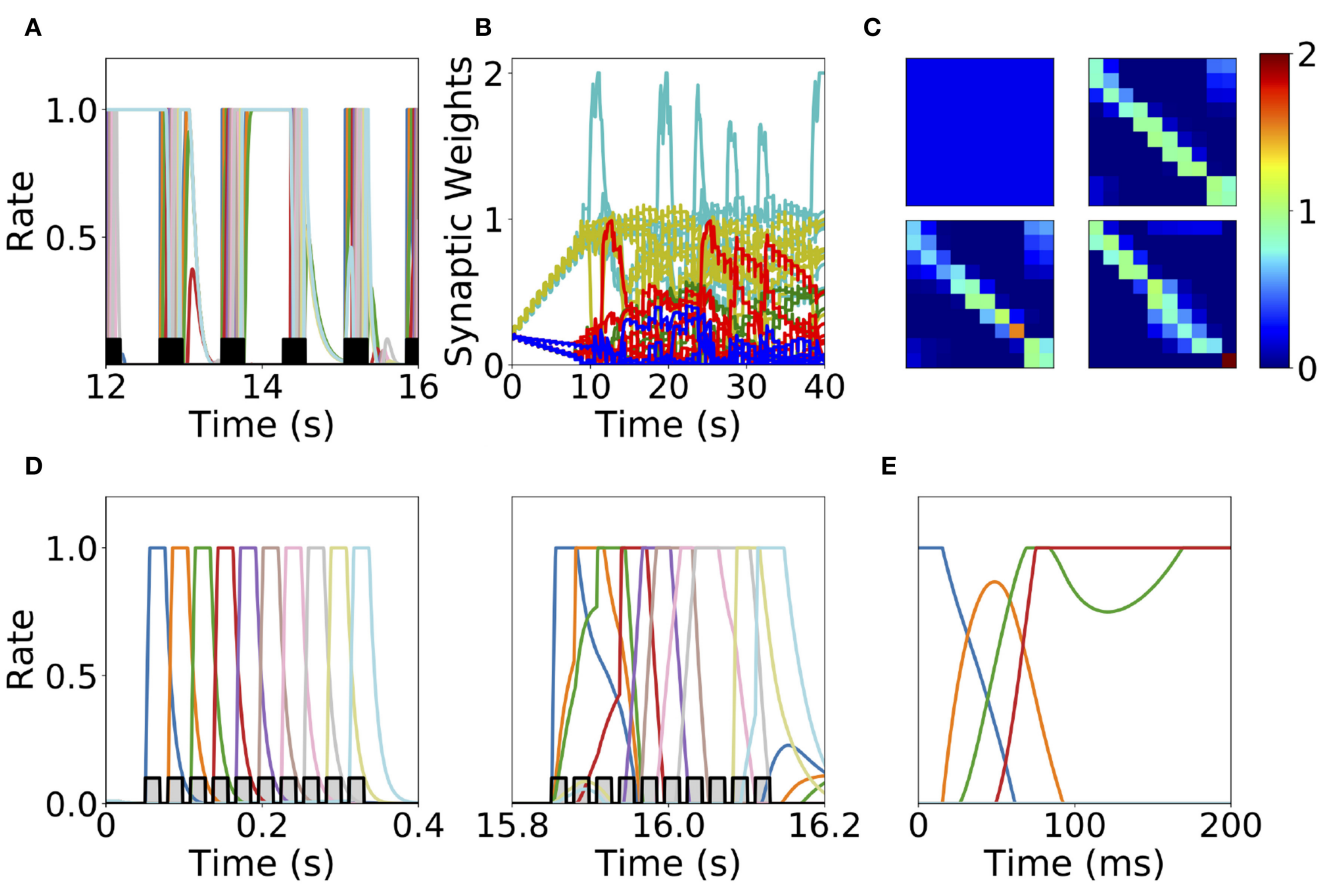
Heterogeneous synaptic dynamics for Hebbian plasticity and synaptic normalization. **(A)** Population dynamics during 10 s of sequential stimulation with *T* = 19 ms and Δ = 10 ms. **(B)** Synaptic weights dynamics during stimulation. Cyan: recurrent connections; Light Yellow/Green: feedforward; Red: feed-backward; Blue: feed-second-forward; Green: feed-second-backward. **(C)** Connectivity matrix at different stimulation times. From left to right and from top to bottom: 0, 13.8, 27.6, and 41.5 s. **(D)** Two examples of population dynamics during a single sequential stimulation at 0 and 15.8 s respectively. In **(A,D)** the black and gray traces indicate a scaled version of the stimulus. **(E)** Network dynamics after learning for the initial condition where the first population is active at high rate and the rest silent. Parameters can be found in Tables S3, S4. Firing rates of excitatory populations are colored according to their position in the sequential stimulation.

The first mechanism we consider is synaptic normalization. This mechanism is motivated by experimental evidence of conservation of total synaptic weight in neurons (Royer and Paré, 2003; Bourne and Harris, 2011). In our model, we enforce that the sum of the incoming synaptic weights to a given population is fixed throughout the dynamics (see Equation 11 in Methods). This constraint prevents the growth of all the synaptic weights to their maximum value during sequential stimulation due to the Hebbian plasticity, as is described in the previous section. This leads to a heterogeneous dynamics in the synaptic weights where they strongly fluctuate in time during the stimulation period (see Figure 7B). We find that the network does not reach a stable connectivity structure, and that the connectivity after the stimulation markedly depends on the specific moment when stimulation ended for a large range of stimulation parameters.

At the initial stages of the stimulation, feedforward and recurrent connections grow, while the rest of the synaptic connections decrease at the same rate (see Figure 7B). When the feedforward and recurrent connections are large enough for producing persistent activity, co-activation between a population(s) undergoing persistent activity and the population active due to the stimulation (which are not necessarily adjacent in the stimulation sequence, see Figures 7A,D) produces an increase in feed-back and upper triangular connections that are different than feedforward and recurrent (see Figure 7B). In turn, feedforward and recurrent connections decrease due to the synaptic normalization mechanism. This leads to complex dynamics in the synaptic weights, in which the connections sustaining co-active neuronal assemblies (i.e., group of excitatory populations with clustered connectivity) learned via Hebbian plasticity are depressed due to the interplay between synaptic normalization and sequential stimulation. This then leads to the formation of new assemblies due to the interplay of Hebbian plasticity and sequential stimulation.

During stimulation, the feedforward and recurrent connectivity studied in the first section increase first, leading then in a second stage to connectivities with strong bi-directional connections (see Figure 7C). Persistent activity can not be learned consistently after long times, and most populations are simultaneously active in retrieved states (see Figure 7E). For a very few parameter values compared with the explored parameter space sequential activity is learned (data not shown). Overall, it is unclear whether neural circuits can use the observed complex synaptic dynamics to store retrievable information about the external stimuli. Thus, we find that synaptic normalization is not sufficient in this case to stabilize learning dynamics and to lead to a consistent retrieval of PA or SA. We checked that this finding is robust to changes in parameters, in particular to changes in the sum of incoming synaptic weights [parameter *C* in Equation (11)] and the sequential stimulation magnitude *I*, period *T*, and delay Δ. In the next section we consider a second stabilization mechanism, namely generalized homeostatic plasticity.

### 2.4. Generalized Homeostatic Plasticity

**Figure 8:**
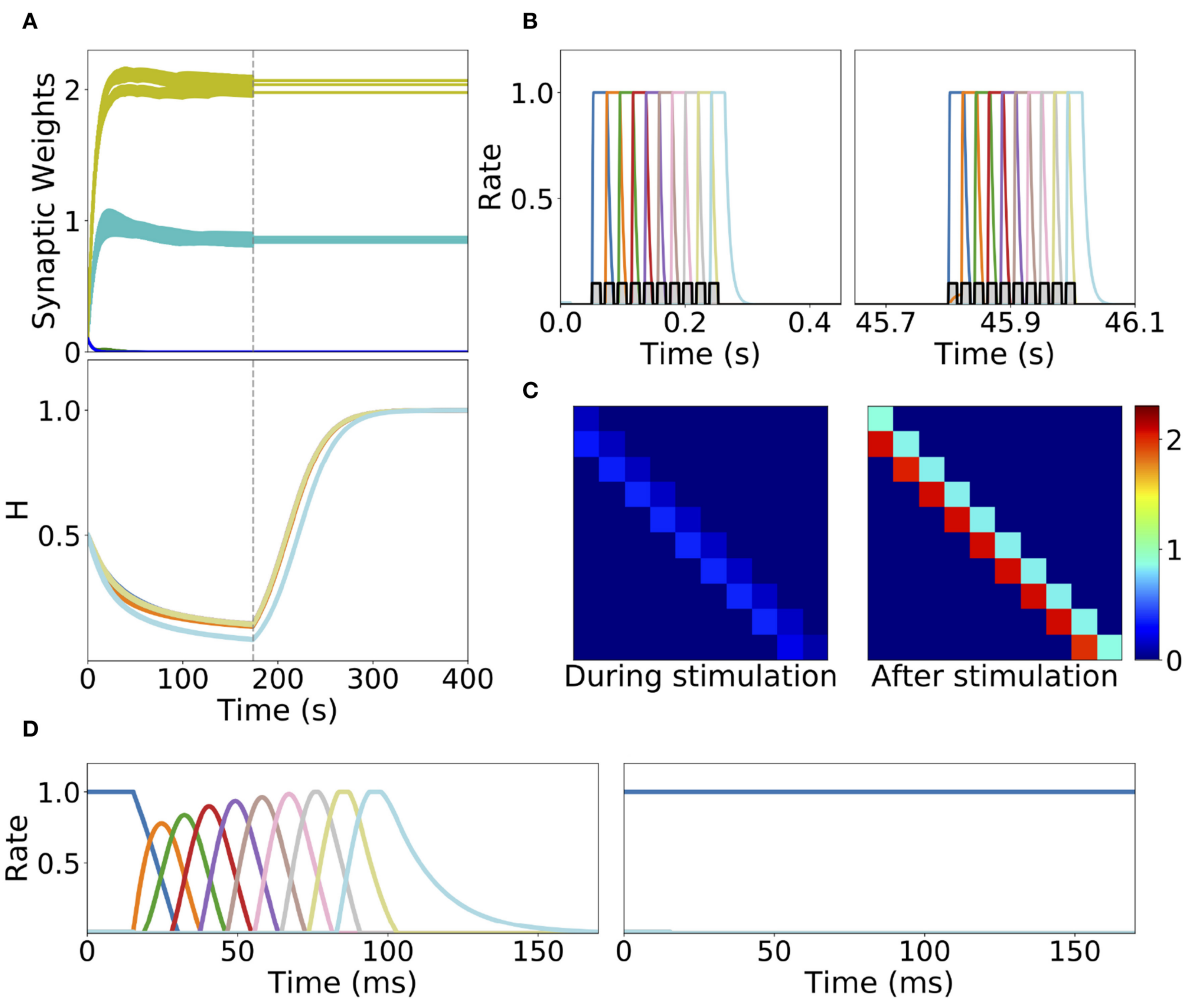
Learning dynamics in a network with Hebbian and generalized homeostatic plasticity. **(A)** Top: synaptic weights dynamics during and after stimulation. Cyan: recurrent; Yellow: feedforward; Blue: all other connections. Bottom: homeostatic variables colored according to the position of the corresponding excitatory population in the sequential stimulation (as in **B**). Gray vertical dashed line indicate the end of the sequential stimulation. **(B)** Neuron dynamics during stimulation for two different periods of time. **(C)** Snapshots of the connectivity matrix **W**_*i,j*_(*t*) at the end of the sequential stimulation (left) and 60 s after the end of the sequential stimulation (right). **(D)** Network dynamics after learning following an initial condition where the first population is active at high rate while all others are silent for two different stimulation parameters, one that generates SA (left), the other PA (right). Parameters can be found in Tables S3, S4. Firing rates of excitatory populations are colored according to their position in the sequential stimulation.

Homeostatic plasticity is another potential stabilization mechanism that has been characterized extensively in experiments (Turrigiano et al., 1998; Turrigiano, 2017). The interplay between homeostatic plasticity and Hebbian plasticity has recently been the focus of multiple theoretical studies (Renart et al., 2003; Toyoizumi et al., 2014; Keck et al., 2017). Here, we study the effect of a generalization of the standard multiplicative homeostatic plasticity and Hebbian plasticity for learning SA and PA. We consider a model for homeostatic plasticity in which the overall connectivity at each time **W**_*i, j*_(*t*) is given by the multiplication of two synaptic variables with different time scales as is shown in Equation (12). In this equation, the fast plastic variable 𝕎_*i, j*_(*t*) (time scale of seconds) is governed by Hebbian plasticity, see Equation (7). On the other hand, the slow (with a time scale of tens to hundred of seconds) homeostatic variable *H*_*i*_(*t*) scales the incoming weights to population *i*, ensuring that the network maintain low average firing rates on long time scales. The dynamics of the homeostatic variable is given by Equation (13). This is a generalization of the standard homeostatic learning rule (Renart et al., 2003; Toyoizumi et al., 2014), that does not include the quadratic term in the r.h.s. of Equation (13). We term this rule generalized homeostatic plasticity since is a non-linear generalization of the standard multiplicative homeostatic plasticity rule in Renart et al. (2003) and Toyoizumi et al. (2014). The equation proposed in Toyoizumi et al. (2014) stabilizes the network's activity during stimulation, preventing the runaway of the firing rates and synaptic weights. Scaling down the overall connectivity during stimulation prevents co-activation of multiple populations, and lead to stable learning. However, in the network's steady state (i.e., when times longer than the time scale of the homeostatic variable have passed without any stimulation), if the equation proposed in Toyoizumi et al. (2014) is used, then each connection will be proportional to the factor $\frac{\phi^{-1}\left(r_{0}\right)}{r_{0}}$ multiplied by a number of order one (see section 5 in the Supplementary Material for the mathematical details). This implies that the steady state connectivity after learning will depend sensitively on the choice of the value of the objective background firing rate (i.e., *r*_0_) and the specific functional form of the transfer function (i.e., ϕ(*u*)). Due to the transfer function non-linearity, small changes in *r*_0_ might produce large values for the factor $\frac{\phi^{-1}\left(r_{0}\right)}{r_{0}}$ and therefore very strong connections for the steady state connectivity. This is due to the fact that steady state large values in the homeostatic variable *H* scale up the connectivity learned via Hebbian plasticity in a multiplicative fashion, see Equation (12). In practice, PA is retrieved almost always independently of the type of stimulation presented during learning, and in the absence of the quadratic term in Equation (13) no temporal attractor other than PA can be learned. This problem can be prevented by the introduction of a quadratic term in the original homeostatic rule (see section 5 in the Supplementary Material). Note that with this quadratic term, the homeostatic plasticity rule does not exactly achieve a given target firing rate, and therefore is not strictly speaking “homeostatic.” Additionally, the timescale of this rule is of the order of minutes (see Figure 8A), which is an order of magnitude faster of what has been reported experimentally (Turrigiano et al., 1998; Turrigiano, 2008, 2017) (see section 3.3 for a discussion).

We explore the role of this generalized homeostatic learning rule for learning both PA and SA. During sequential stimulation, the average firing rate is higher than the background objective firing rate *r*_0_, and the homeostatic variables decrease to values that are smaller than 1, see Figures 8A,C. As a result, during sequential stimulation the dynamics of the homeostatic variable will be dominated by the linear version of the homeostatic learning rule proposed in Toyoizumi et al. (2014), since $H_{i}^{2}\ll 1$. Then, the small values that the homeostatic variables take during the sequential stimulation scale down the increasing values of the recurrent and feedforward connections due to Hebbian plasticity. This produces a weak excitatory connectivity during a repeated sequential stimulation (see Figure 8C, left), preventing activation of spurious populations during stimulation (see Figure 8B), even though the strength of recurrent and feedforward connections learned via Hebbian plasticity are strong enough to produce PA or SA, since these connections are *masked* by the homeostatic variable. When the network returns to the steady state after sequential stimulation, the homeostatic variables return to values $H_{i}\sim O\left(1\right)$ (see section 5 in the Supplementary Material for the mathematical details), and the recurrent and feedforward connections learned via Hebbian plasticity are *unmasked*. This mechanism stabilizes learning, allowing the network to stably learn strong recurrent and feedforward connections, consistent with SA or PA dynamics (see Figure 8D).

**Figure 9:**
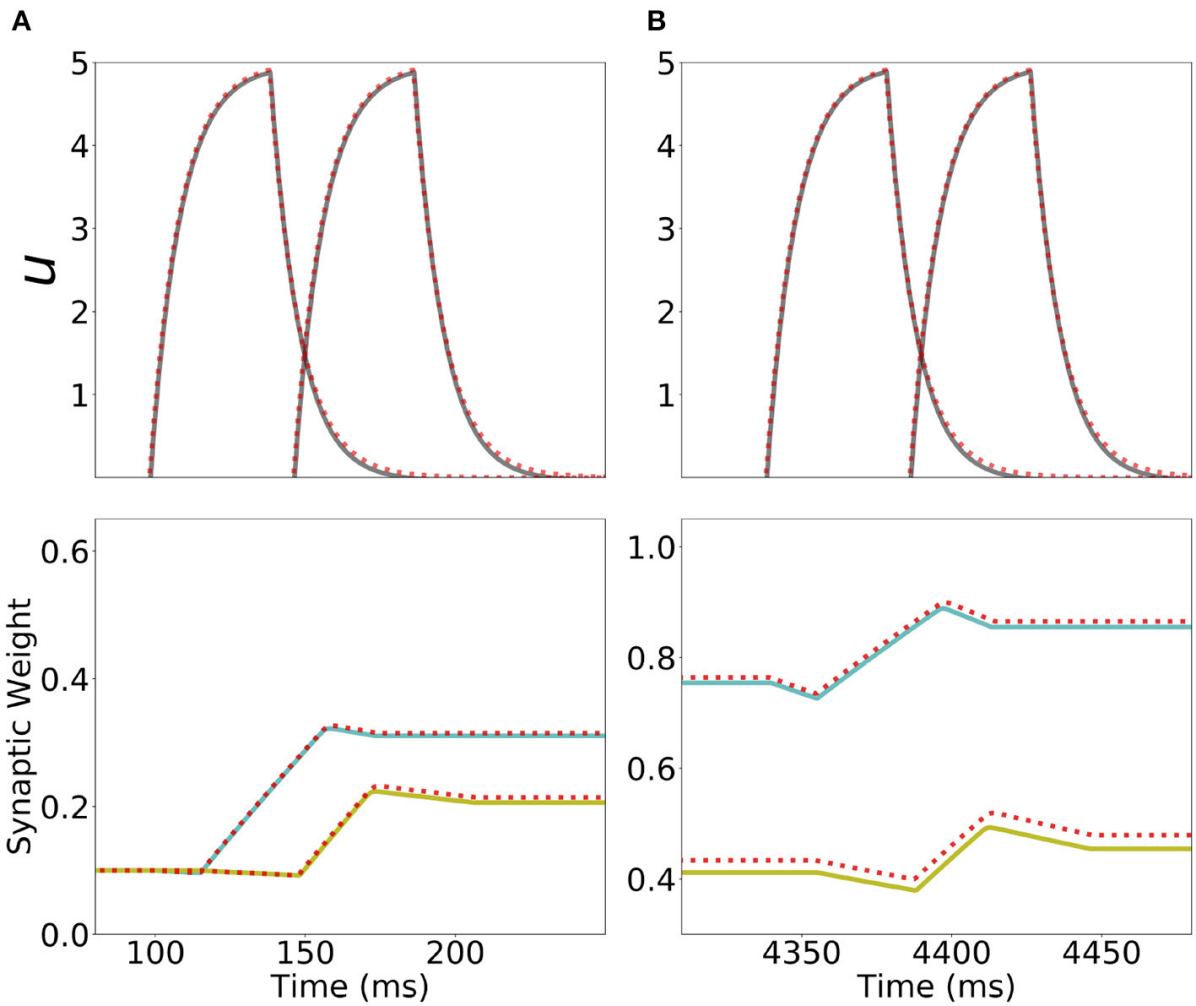
Analytical approximation of the dynamics of the network with Hebbian and generalized homeostatic plasticity. First row: Current dynamics for the second and third populations in a network of 20 populations during one presentation of the sequence. The dashed red line shows the analytical approximation for the dynamics during stimulation (Equation S32 in section 6 of Supplementary Material). Second row: Dynamics of the recurrent synaptic strength within the second population (cyan), and the “feedforward” synaptic strength from the second to the third population (yellow) during the same presentation of the sequence. The dashed red line shows the analytical approximation for the synaptic weight dynamics (Equations S34, S37 in section 6 of Supplementary Materials). **(A,B)** correspond to the first and the fifth presentation of the stimulation sequence respectively. Parameters can be found in Tables S3, S4.

**Figure 10:**
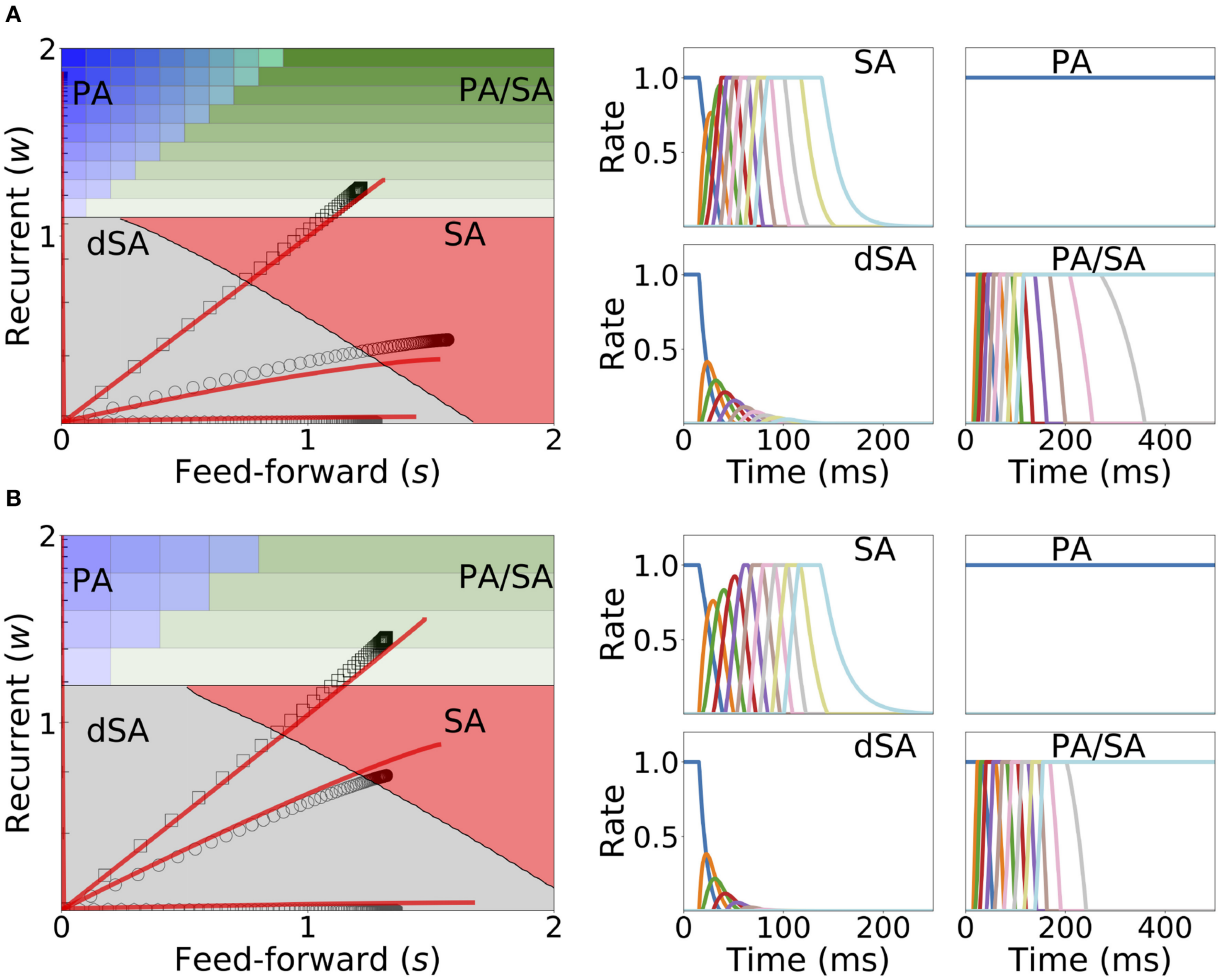
Changes in recurrent and feedforward synaptic strengths with learning, for different sequences with different temporal parameters. Left: Dynamics of recurrent and feedforward connections in the (*s, w*) parameter space during sequential stimulation for four different values of Δ and *T*. Black circles (SA), plus signs (PA), hexagons (dSA), and squares (PA/SA) show the simulated dynamics. Red traces indicate the approximated dynamics derived in section 6 of Supplementary Material. Right: Rates dynamics after many presentations of the sequence. The first population was initialized at high rates, the others at low rates. **(A,B)** correspond to *w*_*I*_ = 1, *w*_*max*_ = 3, and (*T*, Δ) = {(7, 14), (50, 40), (5, 15), (22, 8.5)} (in ms) and *w*_*I*_ = 2, *w*_*max*_ = 3.5, and (*T*, Δ) = {(11, 14), (50, 40), (5, 15), (23, 8.5)}, respectively. The rest of the parameters can be found in Tables S3, S4. Firing rates of excitatory populations are colored according to their position in the sequential stimulation.

The weakening of recurrent connections during sequential stimulation allows us to derive an approximate analytical description of the temporal evolution of the synaptic connectivity with learning. Since the net current due to connections between populations is very small, each population dynamics is well-approximated by an exponential rise (decay) toward the stimulation current (background current) provided the stimulation is strong and inhibition is weak enough (see Figure 9). By using this approximation we build a mapping that yields the value of the recurrent and feedforward synaptic strengths as a function of stimulation number *k*, stimulation period, *T*, and delay, Δ (see Equations S39, S40 in 6 of Supplementary Material). This mapping provides a fairly accurate match of both the dynamics of the synaptic weights and the final steady state connectivity matrix in the case of weak (see Figure 10A, corresponding to *w*_*I*_ = 1) and stronger inhibition (see Figure 10B, *w*_*I*_ = 2). The mapping derived for evolution of the synaptic weights during sequential stimulation corresponds to a dynamical system in the (*s, w*) phase space that depends on the stimulus parameters (Δ, *T*) and the initial connectivity. The final connectivity corresponds to the fixed point of these dynamics (see Equations S41, S42 in section 6 of Supplementary Material).

Figure 10 shows that depending on the temporal characteristics of the input sequence, the network can reach any of the four qualitatively different regions of the phase diagrams in a completely unsupervised fashion. For values of Δ that are smaller than the synaptic delay *D* and *T* on the order or larger than *D*, the network generates SA. For values of *T* approximately larger than *D* and for Δ small enough, the dynamics lead to SA/PA. Lastly PA is obtained for large enough Δ and *T*. These observations match with the intuition that stimulations long enough but far delayed in time leads to learning of PA and that stimulations contiguous in time but short enough leads to SA. Stimulations between these two conditions (long and contiguous) leads to a combination of both dynamics, i.e., SA/PA, as shown in Figure 10.

### 2.5. Learning and Retrieval Is Robust to Noise

**Figure 11:**
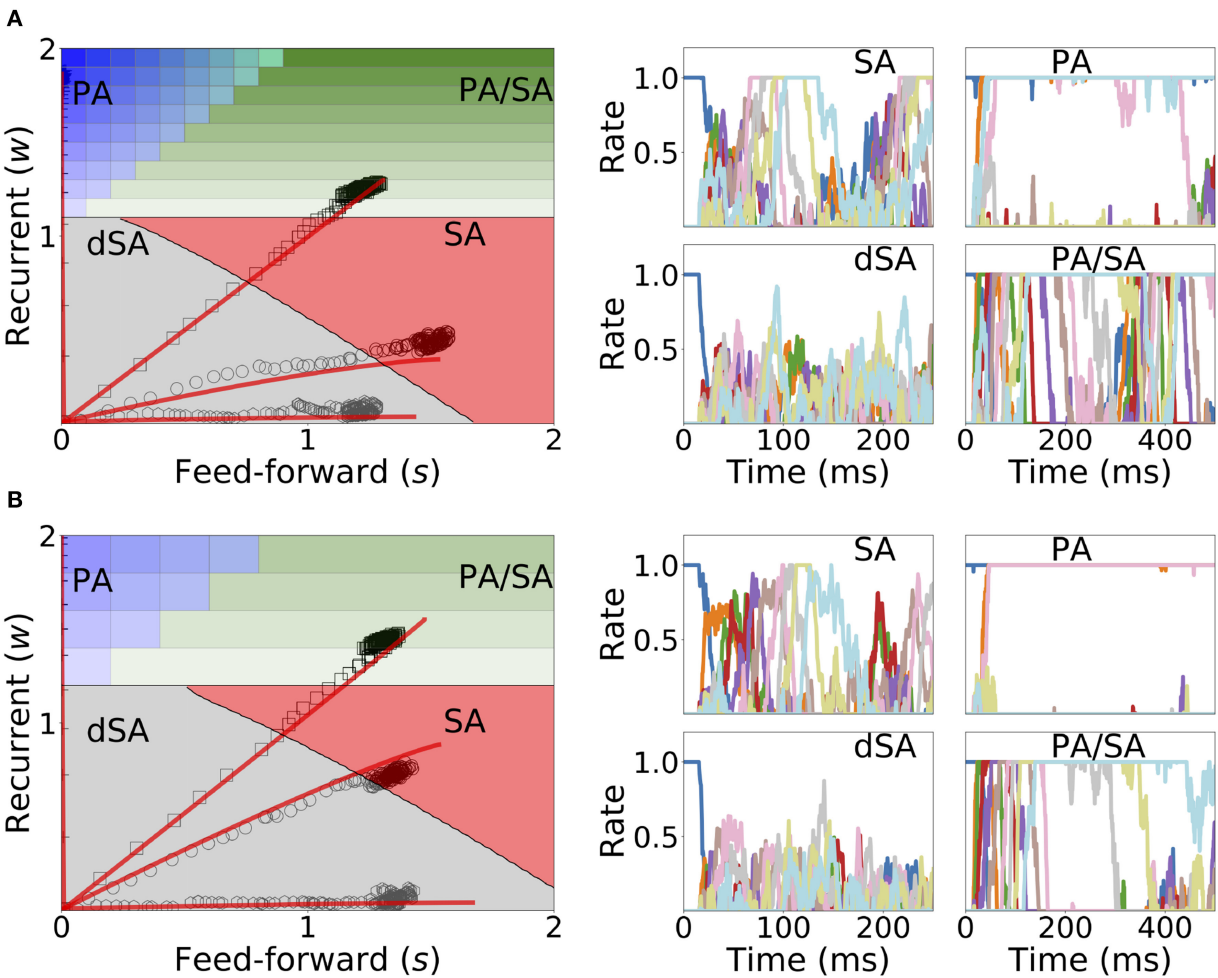
Learning dynamics under noisy stimulation. Same as in Figure 10, but in the presence of a white noise input current, with mean 0 and standard deviation of 0.3 [i.e., σ = 0.3 in Equation (14)]. Firing rates of excitatory populations are colored according to their position in the sequential stimulation.

Under *in vivo* conditions neural systems operate with large amount of variability in their inputs. In order to assess the effect of highly variable synaptic input current during learning and retrieval, we add a mean zero uncorrelated white noise to the dynamics when both Hebbian learning and generalized homeostatic plasticity are included in the network, as described in Equation (14). We found that both the synaptic weights dynamics during learning and the retrieved spatiotemporal dynamics after learning are robust to noise (see Figure 11), even when the amplitude of the noise is large (i.e., inputs with values equal to the standard deviation of the noise lead to a population to fire at 30% of the maximum firing rate). During sequential stimulation, the learning dynamics is marginally altered for both weak and strong inhibition (compare Figure 11 with Figure 10). Importantly, the synaptic weights reach very similar stationary values compared with the case without noise. After learning, even though the rates stochastically fluctuate in time, the retrieved spatiotemporal attractors (i.e., PA, SA, dSA, or PA/SA) are qualitatively similar as in the case without noise (compare Figure 11 with Figure 10). One qualitative difference in the case with external noise, is that in both SA and PA/SA dynamical regimes random inputs lead to a repetition of the full or partial learned sequence. Altogether, this simulations show that the network can robustly learn and retrieve qualitatively the same spatiotemporal attractors in the presence of external noise.

## 3. Discussion

We have shown that under sequential stimulation a network with biologically plausible plasticity rules can learn both PA or SA depending on the stimulus parameters. Two plasticity mechanisms are needed: (1) Hebbian plasticity with temporal asymmetry; (2) a stabilization mechanism which prevents the runaway of synaptic weights while learning. When unsupervised Hebbian plasticity is present alone the network fails to stably learn PA or SA, while including a generalized homeostatic plasticity stabilizes learning. For stable learning, we show that the learning process is described by a low dimensional autonomous dynamical system in the space of connectivities, leading to a simplified description of unsupervised learning of PA and SA by the network from external stimuli. Depending on the stimulus parameters, the network is flexible enough to learn selectively both types of activity by repeated exposure to a sequence of stimuli, without need for supervision. This suggests that cortical circuits endowed with a single learning rule can learn qualitatively different neural dynamics (i.e., persistent vs. sequential activity) depending on the stimuli statistics.

Using the full characterization of the bifurcation diagram in the space of fixed feedforward and recurrent connections developed here, we mapped the evolution of the connectivity during stimulation in the bifurcation diagram. We analytically and numerically showed that the synaptic weights evolve in the feedforward—recurrent synaptic connections space until they reach their steady state (when the number of sequential stimulations is large). The specific point of the steady state in the bifurcation diagram depends solely on the stimulation parameters—stimulation period *T* and time delay Δ—and the connectivity initial conditions. We found that stimulations with long durations and large delays generically leads to the formation of PA, whereas stimulations with long enough durations and short delays leads to the formation of SA. Thus, persistent stimulation leads to persistent activity while sequential stimulation leads to sequential activity.

### 3.1. Learning of Sequences in Networks

A growing number of network models have been shown to be able to learn sequential activity. Models with supervised learning can reproduce perfectly target sequences through minimization of a suitable error function (Sussillo and Abbott, 2009; Laje and Buonomano, 2013; Memmesheimer et al., 2014; Rajan et al., 2016), but the corresponding learning rules are not biophysically realistic.

Other investigators have studied how unsupervised learning rules leads to sequence generation. Early models of networks of binary neurons showed how various prescriptions for incorporating input sequences in the connectivity matrix can lead to sequence generation (see Kuhn and van Hemmen, 1991)—or, sometimes, both sequence generation or fixed point attractors depending on the inputs (Herz et al., 1988). The drawback of these models is that they separated a learning phase in which recurrent dynamics was shut down in order to form the synaptic connectivity matrix, and a retrieval phase in which the connectivity matrix does not change anymore.

Our model removes this artificial separation, since both plasticity rule and recurrent dynamics operate continuously, both during learning and recall. However, we found that there needs to be a mechanism to attenuate recurrent dynamics during learning for it to be stable. The mechanism we propose rely on a modified version of a standard homeostatic rule. Other mechanisms have been proposed, such as neuromodulators that would change the balance between recurrent and external inputs during presentation of behaviorally relevant stimuli (Hasselmo, 2006).

The cost of not having supervision is that the network can only learn the temporal order of the presented stimuli, but not their precise timing. Veliz-Cuba et al. (2015) have recently provided a model which bear strong similarities with our model (rate model with unsupervised temporally asymmetric Hebbian plasticity rule), but includes in addition a short-term facilitation mechanism that allows the network to learn both order and precise timing of a sequence presented in input. However, their mechanisms require parameters to be precisely related.

Models with temporally asymmetric Hebbian plasticity have also been investigated in the context of the hippocampus (Abbott and Blum, 1996; Gerstner and Abbott, 1997; Mehta et al., 1997; Jahnke et al., 2015; Chenkov et al., 2017; Theodoni et al., 2018). In such models, feedforward connectivity is learned through multiple visits of neighboring place fields, and sequential activity (“replays”) can be triggered using appropriate inputs mimicking sharp-wave ripples. Other models use unsupervised Hebbian plasticity but qualitatively distinct mechanisms to generate sequential activity. In particular, several studies showed that sequences can be generated spontaneously from unstructured input noise (Fiete et al., 2010; Okubo et al., 2015). Murray and Escola (2017) showed that sequences can be generated in networks of inhibitory neurons with anti-Hebbian plasticity, and proposed that this mechanism is at work in the striatum.

### 3.2. Learning of Persistent Activity With Unsupervised Hebbian Plasticity

In our model, unsupervised temporally asymmetric Hebbian plasticity is unstable, and after a number of stimulations, all synaptic weights increase to the maximum value. The destabilization occurs during sequential stimulation when the population previously stimulated in the sequence presents persistent activity at the moment the next population is being stimulated. This leads to the increase of the feedforward synaptic weight between these two populations, producing that both present PA while the next population in the sequence is being stimulated. This process continues in each repetition of the sequential stimulation until all populations in the network present PA leading to an increase in the synaptic weights to maximum values. However, previous studies have shown that PA could be learned without additional stabilization mechanisms. In Del Giudice and Mattia (2001), Del Giudice et al. (2003), and Mongillo et al. (2005) PA is learnt without any stabilizing mechanism except for short-term depression. These studies have a number of differences with ours, however, we believe the key difference for stable learning PA is that in these models there is PA (in the absence of inputs to the network) below the threshold for LTP induction - something is not currently present in our model since PA is always at the maximal rate. Therefore, unlike our model, PA after stimulation does not lead to effective changes in the synaptic connections. Interestingly, they introduced short term depression to stabilize learning by rapidly shutting down high firing rates during stimulation or PA, which resembles the effect of the generalized homeostatic plasticity rule in our model. In Mongillo et al. (2003), connectivities with stable recurrent, feedforward and feedback connections between pairs of populations emerge from unsupervised Hebbian learning without any stabilization mechanism. After learning, when one population in the pair presents PA the other transition to PA stochastically after a period of time due to spike noise. Importantly, as in the previous models discussed above, the learning thresholds are set in such a way that there is no learning when both populations present PA. Additionally, during learning the presentations of stimuli are far apart, and unlike our model learning feedback and feedforward connections are due to persistent activity in one neuron, and the stimulation in the other, followed by a small increase in the synaptic strengths in each presentation. Lastly, another difference with the models above, and with classical models for persistent delay activity (Amit and Brunel, 1997), is that in our model the “background state” is at zero activity. This is an unrealistic feature stemming from the simplified piecewise linear transfer function used in our network (see Equation 5).

### 3.3. Stabilization Mechanisms

Consistent with many previous studies (Dayan and Abbott, 2001), we have shown that a network with unsupervised Hebbian plasticity under sequential stimulation leads to a runaway of the synaptic weights. This instability is due to a positive feed-back loop generated by the progressive increase of network activity leading to a progressive increase in average synaptic strength when PA or SA are being learned. One possible solution for this problem was first proposed in the context of attractor neural network models (Amit et al., 1985; Tsodyks and Feigel'Man, 1988; Amit and Fusi, 1994). In these models, patterns are learned upon presentation during a *learning phase* where synapses are plastic but there is no ongoing network dynamics. After the *learning phase*, the learning of attractors is tested in a *retrieval phase*, where the network dynamics is ongoing but synaptic plasticity is not present. Therefore, by compartmentalizing in time dynamics and learning, the network dynamics does not lead to changes in the synaptic weights during retrieval, and conversely, changes in synaptic weights do not lead to changes in the dynamics during learning. This separation prevents the observed runaway of the synaptic weights due to unsupervised Hebbian plasticity.

However, it is unclear whether such compartmentalization exists in cortical networks. In this work, we explored the alternative scenario, in which both plasticity and dynamics happen concurrently during learning and retrieval (see also Mongillo et al., 2005; Litwin-Kumar and Doiron, 2014; Zenke et al., 2015 for a similar approach in networks of spiking neurons). First, we found that by adding to unsupervised Hebbian plasticity an instantaneous synaptic normalization mechanism that maintains constant the sum of incoming synaptic weights to each population, PA and SA cannot be stably learned. Second, we found that adding a generalized homeostatic plasticity to unsupervised Hebbian plasticity leads to stable learning of PA and SA. During sequential stimulation, the increase in co-activation between multiple populations due to recurrent and feedforward connections learned via unsupervised Hebbian plasticity is prevented by suppressing its effect in the network dynamics. Homeostatic plasticity scales down the overall connectivity producing a weakly connected network. PA and SA is prevented to occur during stimulation, which weakens the positive feed-back loop generated by the increase in co-activations of neuronal populations. After learning, the dynamic variables of the Homeostatic plasticity rule reach a steady state with values similar of what they where before stimulation (see Figure 8A) and the connectivity learned via unsupervised Hebbian plasticity can lead to retrieval of PA and SA upon stimulation (see Figure 8C). The homeostatic variable reaches its steady state at a value close to one, and the connectivity recovers, *unmasking* the feedforward and recurrent learned architecture. We have also tried other stabilization mechanisms such as inhibitory to excitatory plasticity (Vogels et al., 2011) instead of homeostatic plasticity. In this case we found that stable learning of PA and SA is possible, but for distinct sets of network and stimulation parameters (data not shown).

As explained in Zenke and Gerstner (2017) and Zenke et al. (2017), in order to prevent the runaway of the synaptic weights produced by Hebbian plasticity, the timescale of any compensatory mechanism should be of the same order or faster than the Hebbian timescale. For generalized homeostatic plasticity, the timescale of the homeostatic variable *H*_*i*_ is dependent on the firing rate of neuron *i* and the target firing rate [i.e., ϕ(*u*_*i*_)/ϕ(*u*_0_)]. When the network firing rate is close to the target firing rate the homeostatic learning rule is slow, and the homeostatic mechanism seldom play a role in the dynamics. On the other hand, for high firing rates the homeostatic plasticity timescale becomes faster, preventing the runaway of the synaptic weights. There is currently an ongoing debate about whether the timescales of compensatory processes used in theoretical studies, as the ones used here, are consistent with experimental evidence (see e.g., Zenke and Gerstner, 2017; Zenke et al., 2017).

## Author Contributions

UP and NB designed, performed the research, and wrote the manuscript.

### Conflict of Interest

The authors declare that the research was conducted in the absence of any commercial or financial relationships that could be construed as a potential conflict of interest.
