# Supplementary material for: Unsupervised Learning of Persistent and Sequential Activity
Source: Front Comput Neurosci. 2020 Jan 17;13:97. doi: 10.3389/fncom.2019.00097 (PMC6978734; doi:10.3389/fncom.2019.00097)
Supplement: Supplementary file 1 [file Data_Sheet_1.PDF]

Supplementary Material: Unsupervised Learning of Persistent and Sequential Activity

1 PARAMETERS VALUES

For the networks with fixed connectivity, the parameters used in Fig 2 and 3 are summarized in the Table S1 and S2 respectively. For networks with plastic connectivity, the parameters used in Fig 4-10 are summarized in Table S3. The sequential stimulation parameters used in Fig 5-10 are summarized in Table S4.

Table S1. Parameters used in Fig 2.

	**(A)**	**(B) I**	**(B) II**
n	14	20	20
w	-	0.05	0.35
s	-	0.6	0.6
τ	10ms	10ms	10ms
w_{EI}	-	-	0.08
w_{IE}	-	-	1
τ_I	-	-	5ms
a	-	6	6
b	-	-0.25	-0.25
ν	1	2	2
θ	0	0	0
u_c	1	0.5	0.5
$\tilde{\nu}$	-	0.8	0.8
$\tilde{\theta}$	-	-0.1	-0.1
$\tilde{u}_c$	-	0.5	0.5

Table S2. Parameters used in Fig 3. In all cases, $n = 10$ and $w_I = 2$.

	**dSA**	**SA**	**SA/PA**	**PA (bottom)**	**PA (top)**
w	0.2	1.01	1.3	1.9	1.3
s	1.5	1.39	1.	0.7	0.15

Table S3. Network parameters used in Fig 4-10. *Values of all the entries in the initial matrix $\mathbb{W}$.

	**Fig 4**	**Fig 5**	**Fig 6**	**Fig 7**	**Fig 8**	**Fig 9**	**Fig 10 (A/B)**
n	10	10	10	10	10	10	10
w_I	1	1	1	1	4.3	1	1/2
w_{max}	1.5	1.5	2.5	2.5	3.5	2.4	3/3.5
T_w	-	400ms	400ms	400ms	400ms	400ms	400ms
$\mathbb{W}_{i,j}(0)$*	-	0.1	0.1	0.2	0.1	0.1	0.1
a_{pre}	10	10	10	10	10	10	10
b_{pre}	0.7	0.7	0.7	0.7	0.7	0.7	0.7
a_{post}	10	10	10	10	10	10	10
b_{post}	0.7	0.7	0.7	0.7	0.7	0.7	0.7
D	-	15.3ms	15.3ms	15.3ms	15.3ms	15.3ms	15.3ms
r_w	0.6	0.6	0.6	0.6	0.6	0.6	0.6
r_0	-	-	-	-	0.05	0.01	0.01
τ_H	-	-	-	-	20s	20s	20s
$\sum_{j=1}^{n} \mathbb{W}_{i,j}$	-	-	-	2	-	-	-

Table S4. Stimulation parameters Fig 4-10.

	I	T (ms)	Δ (ms)
**Fig 5** (top-left)	1.6	18	8
**Fig 5** (top-right)	1.8	20.5	80
**Fig 5** (bottom-left)	1.8	7	9
**Fig 5** (bottom-right)	1.8	1	50
**Fig 6**	1.3	19	10
**Fig 7**	2.2	19	10
**Fig 8** A-C and D (left)	5.4	14	7
**Fig 9**	5	40	8
**Fig 10** (A top-left)	7	7	14
**Fig 10** (A top-right)	7	50	40
**Fig 10** (A bottom-left)	7	5	15
**Fig 10** (A bottom-right)	7	22	8.5
**Fig 10** (B top-left)	7	11	14
**Fig 10** (B top-right)	7	50	40
**Fig 10** (B bottom-left)	7	5	15
**Fig 10** (B bottom-right)	7	23	8.5

2 BIFURCATION DIAGRAM FOR A NETWORK OF EXCITATORY NEURONS WITH RECURRENT AND FEED-FORWARD CONNECTIVITY

Let us consider a network composed of an arbitrary number of excitatory populations. For the sake of analytical tractability, we will use the piecewise linear transfer function, see Eq (5). The goal of this section is to derive the conditions for an initial stimulus to the first population to: 1) propagate throughout the network without decaying; 2) grow until all populations are active at its maximum firing rate; or 3) decay. We consider a stimulus to the network such that all the populations are inactive, except the first (i.e. $\theta \leq u_1(0) \leq u_c$ and $u_j(0) = 0 \quad \forall j \neq 1$). Initially, the inputs to the first population will evolve in time according to

$$u_1(t) = \frac{w\nu\theta}{w\nu - 1} + \left(u_1(0) - \frac{w\nu\theta}{w\nu - 1}\right) e^{-\frac{(1-w\nu)}{\tau}t}.$$

For the sake of simplicity, we choose $\theta = 0$ and $u_1(0) = 1$, and define

$$a \equiv \frac{w\nu - 1}{\tau}$$
$$b \equiv \frac{s\nu}{\tau}.$$

We first compute the dynamics in the linear range of the transfer function, that is, assuming that the inputs are kept within the interval $(0, u_c)$ for the first K populations (i.e. populations k such that $k \leq K \leq n$). The inputs to the first K populations are then given by

$$\tau \frac{du_j}{dt} = -u_j + w\nu u_j + s\nu u_{j-1} \qquad j \leq K, \tag{S1}$$

which leads to

$$u_k(t) = be^{-at} \int_0^t u_k(t') e^{at'} dt' \qquad k \leq K.$$

and therefore

$$u_k(t) = \frac{(bt)^{k-1}}{(k-1)!} e^{at} \qquad k = 1, \ldots, K. \tag{S2}$$

From the above equation we see that if $a > 0$ (i.e. $w > 1/\nu$) and $b > 0$, then the inputs to all populations can only grow in time in the linear range of the transfer function.

On the other hand, if $a < 0$, then Eq. (S2) tells us that inputs to populations $k > 1$ can initially grow, reach a peak and then decay. Provided the dynamics stay in the linear region, inputs to population k peak at time $t_k = -(k-1)/a$. The value of inputs at this peak time is

$$u_{k+1}(t_{k+1}) = \frac{(bk)^k}{(-a)^k k!} e^{-k}.$$

For large k we can use the Stirling approximation for the factorial. This leads to

$$u_{k+1}(t_{k+1}) \sim \frac{1}{\sqrt{2\pi k}} \left(-\frac{b}{a}\right)^k$$

Thus, for $a < 0$ and $-b/a > 1$ (i.e. $w + s > 1/\nu > w$), the peak inputs to populations k grow with k, indicating a stable propagation of a sequence of activations of successive populations. On the other hand, if i.e. $w + s < 1/\nu$ then the peak inputs decays exponentially with k, indicating a failure of propagation.

We can also compute the width of the peak of population activity in the sequence by normalizing the population inputs $u_k(t)$ with their temporal integral ($\int_0^\infty dt u_k(t) = \frac{b^{k-1}}{(-a)^k}$), $\tilde{u}_k(t) \equiv u_k(t) / \left(\frac{b^{k-1}}{(-a)^k}\right)$. The with of the peak of population k is then given by

$$\sqrt{\int_0^\infty t^2 \tilde{u}_k(t) - \left(\int_0^\infty dt \tilde{u}_k(t) t\right)^2} = -\frac{1}{a}\sqrt{k} = \frac{\tau}{1 - w\nu}\sqrt{k}. \tag{S3}$$

Therefore, the time that a population is active in the sequence scales with the squared root of the position of the population in the feed-forward chain (i.e. $\sqrt{k}$, for $k \leq K$). In fact, we found that this scaling also holds for populations whose activity saturate, see Fig 2A.

3 INSTANTANEOUS INHIBITION APPROXIMATION

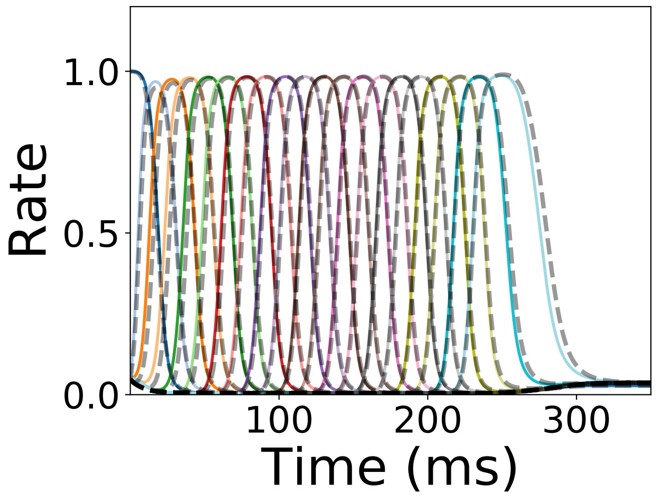

Figure S1. Instantaneous inhibition approximation. Colored lines: SA in a network of 20 populations with fixed feed-forward and recurrent connections and shared inhibition. Grey dashed lines: Instantaneous inhibition approximation. The inhibitory population time constant $\tau_I = 5$ms is half the value of the excitatory populations $\tau = 10$ms. The network parameters are as in Fig 2BII and can be found in Table S1.

4 BIFURCATION DIAGRAM FOR A NETWORK OF EXCITATORY NEURONS WITH RECURRENT AND FEED-FORWARD CONNECTIONS AND SHARED INHIBITION

Here we derive the boundaries between the different regimes of network activity (PA-PA/SA, PA-(SA or dSA), and PA/SA-(SA or dSA)), i.e. the bifurcation diagram of a network of excitatory neurons with recurrent and feed-forward connections and shared inhibition. The dynamics of the network is governed by Eq. (3) of the main text. In addition, we use a piecewise linear transfer function such that $0 \leq \phi(u) \leq 1$ and $u_c = 1$. We first assume that $s = 0$ and that m populations are in a high rate state, i.e. $\phi(u_k) = 1$, whereas all the rest are in the low activity state, i.e. $\phi(u_j) = 0$. If $\phi(u_{k_j}) = 1$ for $k_1, k_2, \cdots, k_m$, a necessary condition for the high rate state to be a fixed point is

$$u_{k_j} = w - \frac{mw_I}{n} > 1 \quad j = 1, \ldots, m. \tag{S4}$$

To have at most m populations in the high rates state as a fixed point, then

$$u_{k_j} = w - \frac{(m+1)w_I}{n} < 1 \quad j = 1, \ldots, m, \tag{S5}$$

needs to be satisfied, which implies

$$1 + \frac{mw_I}{n} \leq w \leq 1 + \frac{(m+1)w_I}{n}. \tag{S6}$$

Let us now consider $s \neq 0$. To have at most r contiguous populations connected via feed-forward connections in the high rate, a necessary condition is that the first population in the chain needs to be able to sustain PA when r populations are active, i.e.

$$u_k = w - \frac{rw_I}{n} > 1. \tag{S7}$$

The second necessary condition is that the last of the r population in the recurrent-feed-forward connected chain does not die out to the low rate state due to inhibition, when this population is in the low activity state and the next population is in high rate state, i.e.

$$u_{k+r-1} = s - \frac{(r-1)w_I}{n} > 0. \tag{S8}$$

To have at most r populations active, we need to impose that the previous population in the chain remains in the low rate state,

$$u_{k+r} = s - \frac{rw_I}{n} < 0, \tag{S9}$$

which is equivalent to

$$\frac{(r-1)w_I}{n} \leq s \leq \frac{rw_I}{n}. \tag{S10}$$

If w fulfills Eq (S6) and we stimulate all the populations of the network, at most m populations remain active. On the other hand, if Eqs. (S7,S8) hold, and we stimulate just the first population in the network

then at least r contiguous populations remain persistently active. Last, if we activate the first population and all the rest are in the low activity state, and

$$\frac{rw_I}{n} \leq s \qquad \text{(S11)}$$
$$1 + \frac{rw_I}{n} \leq w \leq 1 + \frac{(r+1)w_I}{n},$$

the next r populations go to the high rate activity state. Due to shared inhibition, the first population decreases to the low rate state, since Eqs. (S11) holds and it is the population that receives less current because it lacks feed-forward inputs. The decrease in the shared inhibitory input due to the activity decay of the first population leads to the $(r+2)^{\text{th}}$ population to increase its activity toward a high rate activity state. Consequently, the second population decays to the low rate state, due to a new increase in shared inhibition. This process continues, producing a sequence that stabilizes when the last population and the $r-1$ populations before the last are in the high rate state. We call SA/PA this sequential activity ending in persistent activity.

5 GENERALIZED HOMEOSTATIC PLASTICITY

The Hebbian component in the synaptic weight matrix is denoted by $\mathbb{W}_{i,j}$. In the following, we assume that the sequential stimulation is in the range of parameters for T and Δ where only recurrent and feed-forward connections change due stimulation. For a fixed Hebbian matrix, using the plasticity rule proposed in Renart et al. (2003); Toyoizumi et al. (2014) (i.e. the linear version of Eq. (13) in the main text) the fixed-point for the noiseless version of the network dynamics in Eq. (14) is given by

$$u_i^* = u_0 \qquad i = 1, \dots, n \qquad \text{(S12)}$$
$$H_1 = \frac{u_0 + w_I \phi(u_0)}{\phi(u_0)\mathbb{W}_{1,1}} \qquad \text{(S13)}$$
$$H_i = \frac{u_0 + w_I \phi(u_0)}{\phi(u_0)(\mathbb{W}_{i,i} + \mathbb{W}_{i,i-1})} \qquad i = 2, \dots, n \qquad \text{(S14)}$$

where the target firing rate is $r_0 = \phi(u_0)$. Then, the connectivity matrix for the excitatory populations is given by

$$\mathbf{W}_{1,1} = \left(\frac{u_0}{\phi(u_0)}\right)(1 + w_I) \qquad \text{(S15)}$$
$$\mathbf{W}_{i,i} = \left(\frac{u_0}{\phi(u_0)}\right)\left(\frac{1 + w_I}{1 + \frac{\mathbb{W}_{i,i-1}}{\mathbb{W}_{i,i}}}\right) \qquad i = 2, \dots, n \qquad \text{(S16)}$$
$$\mathbf{W}_{i,i-1} = \left(\frac{u_0}{\phi(u_0)}\right)\left(\frac{1 + w_I}{1 + \frac{\mathbb{W}_{i,i}}{\mathbb{W}_{i,i-1}}}\right) \qquad i = 2, \dots, n. \qquad \text{(S17)}$$

Assuming that the sequential stimulation parameters are such that the recurrent and feed-forward connections have the same order of magnitude

$$\frac{\mathbb{W}_{i,i}}{\mathbb{W}_{i,i-1}} \sim O(1),$$

we obtain

$$\mathbf{W}_{i,i-1} \sim \mathbf{W}_{i,i} \sim O\left(\frac{u_0}{\phi(u_0)}\right). \tag{S18}$$

Therefore, after sequential stimulation, the connectivity matrix is proportional to the ratio of synaptic input current u_0 at the target firing rate, and the corresponding target firing rate $r_0 = \phi(u_0)$. For realistic transfer functions ϕ is sublinear for low firing rates. Therefore, the ratio $\frac{u_0}{\phi(u_0)}$ diverges as the target firing rate approaches to zero.

In the case of the generalized homeostatic learning rule in Eq. (13) in the main text, the fixed-points are given by

$$\frac{\phi(u_1^*)}{r_0} = 1 - \frac{u_1^* + \frac{w_I}{n}\sum_{j=1}^n \phi(u_j^*)}{\mathbb{W}_{1,1}\phi(u_1^*)} \tag{S19}$$

$$\frac{\phi(u_i^*)}{r_0} = 1 - \frac{u_i^* + \frac{w_I}{n}\sum_{j=1}^n \phi(u_j^*)}{\mathbb{W}_{i,i-1}\phi(u_{i-1}^*) + \mathbb{W}_{i,i}\phi(u_i^*)} \qquad i = 2, \ldots, n \tag{S20}$$

$$H_1 = \frac{u_1^* + \frac{w_I}{n}\sum_{j=1}^n \phi(u_j^*)}{\mathbb{W}_{1,1}\phi(u_1^*)} = 1 - \frac{\phi(u_1^*)}{r_0} \tag{S21}$$

$$H_i = \frac{u_i^* + \frac{w_I}{n}\sum_{j=1}^n \phi(u_j^*)}{\mathbb{W}_{i,i-1}\phi(u_{i-1}^*) + \mathbb{W}_{i,i}\phi(u_i^*)} = 1 - \frac{\phi(u_i^*)}{r_0} \qquad i = 2, \ldots, n. \tag{S22}$$

Then, the connectivity matrix for the excitatory populations is given by

$$\mathbf{W}_{i,i} = \mathbb{W}_{i,i}\left(1 - \frac{\phi(u_i^*)}{r_0}\right) \qquad i = 1, \ldots, n \tag{S23}$$

$$\mathbf{W}_{i,i-1} = \mathbb{W}_{i,i-1}\left(1 - \frac{\phi(u_i^*)}{r_0}\right) \qquad i = 2, \ldots, n. \tag{S24}$$

If the fractions in the right hand side of Eqs. (S19,S20) are such that

$$0 < \frac{u_1^* + \frac{w_I}{n}\sum_{j=1}^n \phi(u_j^*)}{\mathbb{W}_{1,1}\phi(u_1^*)} < 1 \tag{S25}$$

$$0 < \frac{u_i^* + \frac{w_I}{n}\sum_{j=1}^n \phi(u_j^*)}{\mathbb{W}_{i,i-1}\phi(u_{i-1}^*) + \mathbb{W}_{i,i}\phi(u_i^*)} < 1 \quad i = 2, \ldots, n, \tag{S26}$$

and are order one, i.e.,

$$\frac{u_1^* + \frac{w_I}{n}\sum_{j=1}^{n}\phi(u_j^*)}{\mathbb{W}_{1,1}\phi(u_1^*)} \sim O(1) \tag{S27}$$

$$\frac{u_i^* + \frac{w_I}{n}\sum_{j=1}^{n}\phi(u_j^*)}{\mathbb{W}_{i,i-1}\phi(u_{i-1}^*) + \mathbb{W}_{i,i}\phi(u_i^*)} \sim O(1) \quad i = 2,\dots,n, \tag{S28}$$

which implies $0 < \left(1 - \frac{u_i^*}{\phi(u_i^*)}\right) \sim O(1)$. Then we have

$$\frac{\mathbf{W}_{ij}}{\mathbb{W}_{ij}} \sim O(1).$$

In this parameter regime, the connectivity in the steady state is proportional to the connectivity learned via Hebbian plasticity by a factor order 1. Therefore, the synaptic weights learned via Hebbian plasticity affect the network dynamics after learning if they are strong enough.

6 APPROXIMATION FOR THE SYNAPTIC WEIGHTS DYNAMICS DURING REPEATED SEQUENTIAL STIMULATION

In this section we obtain an approximation for the synaptic weight dynamics during the sequential stimulation protocol for a network with Hebbian and generalized homeostatic plasticity. First, we compute the changes in synaptic weights after a single stimulation by using an approximation of the time the neuron's current u_i is above the learning threshold $u_w \equiv \phi^{-1}(r_w)$. During the sequential stimulation protocol, the effective connectivity is very weak due to the generalized homeostatic plasticity (i.e. $\mathbf{W}_{i,j} = H_i\mathbb{W}_{i,j} \ll 1$). Then, neglecting the effect of inhibition, the dynamics of each population can be approximated by

$$\tau\dot{u}_i \approx I - u_i \quad i = 1,\dots,n.$$

During the stimulation period, the synaptic input currents are given by

$$u_i(t) = I\left(1 - e^{-\frac{t}{\tau}}\right) \quad t \in [0, T],$$

and their final value right after the stimulation is

$$u_i(T) = I(1 - e^{-\frac{T}{\tau}}).$$

After the stimulation the input currents decay as

$$u_i = u_i(T)e^{-\frac{t}{\tau}} = I(1 - e^{-\frac{T}{\tau}})e^{-\frac{t}{\tau}}.$$

Then, the approximate time that each population takes to reach the learning threshold from resting is

$$\tau_{u_0,u_w} \equiv -\tau \ln\left(1 - \frac{u_w}{I}\right), \tag{S29}$$

while the approximate time that each population takes to decay to the learning threshold from its maximum activity after stimulation ($u_{max} = I(1 - e^{-\frac{T}{\tau}})$) is given by

$$u_w = I(1 - e^{-\frac{T}{\tau}})e^{-\frac{\tau_{u_{max},u_w}}{\tau}},$$

which leads to

$$\tau_{u_{max},u_w} = -\tau \ln \left(\frac{u_w}{I\left(1 - e^{-\frac{T}{\tau}}\right)} \right). \tag{S30}$$

Hence, an approximation for the time that each population spends above the learning threshold is

$$\tau_{u_w} = T - \tau_{u_0,u_w} + \tau_{u_{max},u_w} \tag{S31}$$

The dynamics of above the plasticity threshold u_w when is stimulated at time t_k can be approximated by

$$u_i(t - t_k) = \begin{cases} I\left(1 - e^{-\frac{t-t_k}{\tau}}\right) & t - t_k \in [\tau_{u_0,u_w}, T] \\ I(1 - e^{-\frac{T}{\tau}})e^{-\frac{t-t_k}{\tau}} & t - t_k \in [T, \tau_{u_w}] \end{cases} \tag{S32}$$

Let us first consider the change in recurrent connections. First define $\tilde{t_k} \equiv t - t_k$ and

$$\Omega_{i,j}(a, b) = f[\phi(u_i(a)))]g[\phi(u_j(b - D))].$$

In order to compute the increment in the synaptic weight $\mathbb{W}_{i,i}$ we need to solve

$$\frac{d\mathbb{W}_{i,i}}{d\tilde{t_k}} = \frac{\Omega_{i,i}(\tilde{t_k}, \tilde{t_k} - D) - \mathbb{W}_{i,j}}{\tau_w}, \tag{S33}$$

for

$$\tilde{t_k} \in [\tau_{u_0,u_w}, T + \tau_{u_{max},u_w} + D].$$

We obtain

$$\mathbb{W}_{i,i}(\tilde{t_k}) = e^{-\frac{\tilde{t_k}-\tau_{u_0,u_w}}{\tau_w}} \left(\mathbb{W}_{i,i}(\tau_{u_0,u_w}) + \frac{1}{\tau_w} \int_{D+\tau_{u_0,u_w}}^{\tilde{t_k}} dt\Omega_{i,i}(t_k, t_k - D)e^{\frac{t-\tau_{u_0,u_w}}{\tau_w}} \right), \tag{S34}$$

for

$$\tilde{t_k} \in [\tau_{u_0,u_w}, T + \tau_{u_{max},u_w} + D].$$

To compute the change in feed-forward connections after one stimulation, let us define $\tilde{t_k^i} \equiv t - t_k^i$ as the time elapsed after stimulation of neuron i. In order to compute the increment in the synaptic weight $\mathbb{W}_{i+1,i}$ we need to solve the following equation

$$\frac{d\mathbb{W}_{i+1,i}}{d\tilde{t_k^{i+1}}} = \frac{\Omega_{i+1,i}(\tilde{t_k^{i+1}}, \tilde{t_k^i} - D) - \mathbb{W}_{i,j}}{\tau_w}, \tag{S35}$$

By considering the structure of the sequential stimulation, then we have $\tilde{t}_k^{i+1} = \tilde{t}_k^i + \Delta + T$, obtaining

$$\frac{d\mathbb{W}_{i+1,i}}{d\tilde{t}_k^{i+1}} = \frac{\Omega_{i+1,i}(\tilde{t}_k^i - \Delta - T, \tilde{t}_k^i - D) - \mathbb{W}_{i+1,i}}{\tau_w}, \tag{S36}$$

for

$$\tilde{t}_k^i \in [\tau_{u_0,u_w} + D, \tau_{u_{max},u_w} + \Delta + 2T].$$

We then obtain the following approximation for the dynamics of the feed-forward connections

$$\begin{aligned} \mathbb{W}_{i+1,i}(\tilde{t}_k^i) &= e^{-\frac{\tilde{t}_k^i - \tau_{u_0,u_w} - D}{\tau_w}} \mathbb{W}_{i+1,i}(\tau_{u_0,u_w} + D) \\ &+ \frac{e^{-\frac{\tilde{t}_k^i - \tau_{u_0,u_w} - D}{\tau_w}}}{\tau_w} \int_{\tau_{u_0,u_w}+D}^{\tilde{t}_k^i} dt \Omega_{i+1,i}(t - \Delta - T, t - D) e^{\frac{t - \tau_{u_0,u_w} - D}{\tau_w}} \end{aligned} \tag{S37}$$

for

$$\tilde{t}_k^i \in [\tau_{u_0,u_w} + D, \tau_{u_{max},u_w} + \Delta + 2T].$$

These approximations appear to be accurate for the dynamics during stimulation, as Fig 9 shows. Using Eqs. (S34,S37), we can write iterative equations for the final recurrent and feed-forward synaptic weights for sequential stimulation $k+1$. Defining

$$\begin{aligned} \Gamma_l^{rec} &\equiv \tau_{u_0,u_w} \\ \Gamma_u^{rec} &\equiv T + \tau_{u_{max},u_w} + D \\ \Gamma_l^{ff} &\equiv \tau_{u_0,u_w} + D \\ \Gamma_u^{ff} &\equiv \tau_{u_{max},u_w} + \Delta + 2T, \end{aligned}$$

we obtain

$$\begin{aligned} \frac{\mathbb{W}_{i,i}^{k+1}}{e^{-\frac{\Gamma_u^{rec} - \Gamma_l^{rec}}{\tau_w}}} &= \mathbb{W}_{i,i}^k + \frac{\int_{\Gamma_l^{rec}}^{\Gamma_u^{rec}} dt \Omega_{i,i}(t, t - D) e^{\frac{t - \Gamma_l^{rec}}{\tau_w}}}{\tau_w} \\ \frac{\mathbb{W}_{i+1,i}^{k+1}}{e^{-\frac{\Gamma_u^{ff} - \Gamma_l^{ff}}{\tau_w}}} &= \mathbb{W}_{i+1,i}^k + \frac{\int_{\Gamma_l^{ff}}^{\Gamma_u^{ff}} dt \Omega_{i+1,i}(t - \Delta - T, t - D) e^{\frac{t - \Gamma_l^{ff}}{\tau_w}}}{\tau_w}, \end{aligned} \tag{S38}$$

where $\mathbb{W}^k_{i,i}$ and $\mathbb{W}^k_{i+1,i}$ are the recurrent and feed-forward connections after sequential stimulation k. Iterating Eqs. (S38,S39) above we obtain

$$\begin{aligned}\mathbb{W}^{k+1}_{i,i} &= e^{-\frac{k(\Gamma_u^{rec}-\Gamma_l^{rec})}{\tau_w}}\mathbb{W}^0_{i,i} \\ &+ \left(\sum_{j=1}^{k} e^{-\frac{j(\Gamma_u^{rec}-\Gamma_l^{rec})}{\tau_w}}\right)\frac{1}{\tau_w}\int_{\Gamma_l^{rec}}^{\Gamma_u^{rec}} dt\Omega_{i,i}(t,t-D)e^{\frac{t-\Gamma_l^{rec}}{\tau_w}}\end{aligned} \tag{S39}$$

$$\begin{aligned}\mathbb{W}^{k+1}_{i+1,i} &= e^{-\frac{k(\Gamma_u^{ff}-\Gamma_l^{ff})}{\tau_w}}\mathbb{W}^0_{i+1,i} \\ &+ \left(\sum_{j=1}^{k} e^{-\frac{j(\Gamma_u^{ff}-\Gamma_l^{ff})}{\tau_w}}\right)\frac{1}{\tau_w}\int_{\Gamma_l^{ff}}^{\Gamma_u^{ff}} dt\Omega_{i+1,i}(t-\Delta-T,t-D)e^{\frac{t-\Gamma_l^{ff}}{\tau_w}}.\end{aligned} \tag{S40}$$

For a large number of repetitions of the sequential stimulation (i.e., $k \to \infty$) the stationary recurrent and feed-forward connections are given by

$$\begin{aligned}\mathbb{W}^{\infty}_{i,i} &= \frac{1}{\tau_w}\left(\frac{e^{-\frac{\Gamma_u^{rec}-\Gamma_l^{rec}}{\tau_w}}}{1-e^{-\frac{\Gamma_u^{rec}-\Gamma_l^{rec}}{\tau_w}}}\right)\int_{\Gamma_l^{rec}}^{\Gamma_u^{rec}} dt\Omega_{i,i}(t,t-D)e^{\frac{t-\Gamma_l^{rec}}{\tau_w}} \\ \mathbb{W}^{\infty}_{i+1,i} &= \frac{1}{\tau_w}\left(\frac{e^{-\frac{\Gamma_u^{ff}-\Gamma_l^{ff}}{\tau_w}}}{1-e^{-\frac{\Gamma_u^{ff}-\Gamma_l^{ff}}{\tau_w}}}\right)\int_{\Gamma_l^{ff}}^{\Gamma_u^{ff}} dt\Omega_{i+1,i}(t-\Delta-T,t-D)e^{\frac{t-\Gamma_l^{ff}}{\tau_w}}.\end{aligned} \tag{S41}$$
